# On the structural identifiability of nonlinear models of ligand binding dynamics

**DOI:** 10.1007/s10928-026-10040-z

**Published:** 2026-07-18

**Authors:** Carla White, Vivi Rottschäfer, Lloyd Bridge

**Affiliations:** 1 Swansea University, Swansea, United Kingdom; 2 Leiden University, Leiden, Netherlands; 3 University of Amsterdam, Amsterdam, Netherlands; 4 University of the West of England, Bristol, England

**Keywords:** Mathematical pharmacology, Receptor theory, Differential equations, Structural identifiability analysis

## Abstract

Quantification of drug-cell interactions and subsequent cellular responses by using experimental data together with mathematical models of assumed binding and signalling schematics is vital to many research programmes; data fitting provides estimates for important pharmacological parameters including kinetic parameters controlling drug affinity and efficacy. Ordinary differential equation (ODE) models are a key component of many receptor theory studies used for this purpose. In using ODE simulations to fit experimental data and estimate these parameters, the theory of the identifiability properties of the system is often overlooked. Indeed, structural identifiability analysis (SIA) is often overlooked in many fields of bio-modelling. Building on recent SIA for linear ligand binding models in receptor theory, we present a new analysis of identifiability properties of nonlinear receptor theory models. We include models of ligand depletion in binding assays and ligand-induced dimerisation (LID). The classical SIA approaches of Taylor Series and similarity transformation are applied, using detailed step-by-step calculations to illustrate the complexity of the implementations. New results are obtained which show that the nonlinear ligand-depletion counterpart models of non-identifiable linear ligand excess models are globally identifiable from a single timecourse. Also, the LID model is shown to be globally identifiable if an experimental aparatus-dependent parameter is obtained. The analysis highlights issues of tractability of the methods for similar and higher-dimensional nonlinear models in receptor theory.

## Introduction

Mathematical pharmacology has recently emerged as a modern research field [36], with mathematical modelling and analysis of ligand-mediated signalling playing a key role in understanding complex cellular signalling processes, drug distribution mechanisms and datasets of pharmacological importance. Understanding and quantifying the dynamics of drug-cell interactions and potential cellular responses are key aims of modern analytical pharmacology. Mathematical modelling of ligand binding dynamics continues to contribute to the field as new ligand-binding paradigms (including oligomerisation and allosterism) for receptor theory emerge. Analysis of new mathematical models is important towards the potential development of novel therapeutics [2, 37]. A detailed, quantitative understanding of the consequences of receptor dimerisation and higher order oligomerisation in particular is required as such receptors have been identified as being amongst the targets of some of the most promising therapeutic candidates [2].

Quantifying ligand-receptor interactions by applying parameter estimation techniques and using timecourse data is a primary aim of ligand binding assay analysis. Therefore it is important to consider the identifiability properties of the parameters in the mathematical models of binding kinetics. Typically, one analyses ordinary differential equation (ODE) models for binding dynamics and a number of binding schematics [43, 44]. Structural identifiability analysis (SIA) is an often-overlooked element of such ODE modelling studies, however the question of which parameters may be identifiable from a given dataset is crucial [45]. That is, the practical task of parameter estimation should take account of the process of assessing whether it is even theoretically possible to estimate a set of parameters *uniquely* from experimental observations and the dynamic equations [9, 38].

A simple example of a ligand binding ODE model whose parameters are not uniquely identifiable from a single timecourse is the ODE for a ligand (at constant concentration) that binds to monomeric receptors. Hence, consider the reaction schemewhere ligand A binds to receptor R to form the complex AR, and $k_{a+}$ and $k_{a-}$ are the binding and dissociation rate constants respectively. For a known constant ligand concentration [*A*] and total receptor concentration $R_{tot}$, the ODE system for [*R*] and [*AR*] as functions of time yields the solution

1.1 $$\begin{aligned} [AR](t)=\frac{k_{a+}[A]R_{tot}}{k_{a+}[A]+k_{a-}}\left( 1-e^{-(k_{a+}[A]+k_{a-})t}\right) . \end{aligned}$$

Considering a measured output [*AR*](*t*), there are clearly two “identifiable” parameter combinations (see [45]), namely

1.2 $$\begin{aligned} k_{a+}[A]R_{tot}\qquad \text {and}\qquad k_{a+}[A]+k_{a-}. \end{aligned}$$

Hence, the individual rate constants $k_{a+}$ and $k_{a-}$ are not identifiable from a single timecourse output without further information.

Recent work by the authors has established identifiability properties of a number of low-dimensional linear ODE models in receptor theory [45], representing ligand binding kinetics for ligands at monomeric receptors (both single ligand and Motulsky-Mahan competition [23]) and at pre-dimerised G protein-coupled receptors (GPCRs) [43]. In addition to presenting the new identifiability properties of the receptor binding models, the work has provided a tutorial on the application of classical SIA methods (transfer function, Taylor Series and similarity transformation) to linear ODE models in receptor theory, and presented practical approaches to mitigate the problem of non-identifiability [45].

While GPCRs may exist as pre-formed dimers, vascular endothelial growth factor receptors (VEGFRs) are an example of receptors which dimerise in response to ligand binding [19]. In contrast to the GPCR dimer model, the ODE model for ligand induced dimerisation (LID) is nonlinear [44]. VEGFR binding data is seen to fit well to the model, and the recovered parameter estimates are plausible [44], but identifiability properties of this model are yet to be elucidated. For completeness of the proposed SIA-parameter estimation workflow for receptor theory suggested by [45], we now consider SIA applied to the LID model.

For nonlinear models, the transfer function method (which is relatively straightforward to implement for linear models [45]) is not applicable. In this paper, we consider SIA approaches using Taylor Series and similarity transformation methods for nonlinear systems. Following the tutorial style of [45], we first consider a simple, first order nonlinear model for ligand binding (namely *depleting* ligand binding monomeric receptor [21]). In doing so, we find a noteworthy SIA result for ligand depletion. We thus investigate whether the result extends to the case of pre-dimerised receptors. We then establish the identifiability properties of the LID model.

The remainder of this paper is organised as follows. In Section“Background theory and methods”, we present the pertinent theory behind SIA for nonlinear systems. In Section“Ligand binding monomeric receptor with ligand depletion”, we apply the theory to a model of ligand binding at monomeric receptor with ligand depletion. This gives both a demonstration of our computation method and some new, surprising results on the identifiability of a ligand depletion system. We follow this with SIA applied to a new model of ligand depletion at a pre-dimerised receptor in Section“Ligand binding pre-dimerised receptor with ligand depletion”. In Section“Ligand-induced dimerisation (LID)” we proceed to analyse the LID model for identifiability properties. We conclude in Section“Discussion” with a discussion of our main results and contribution to the literature.

## Background theory and methods

We first discuss some of the theoretical underpinnings of SIA and the methods that we will use in the later examples, following the notation as used in [5]. The theory is also introduced in [45], and we take the detailed definitions from [42]. In [1], a number of related and alternative definitions are listed.

We consider biological systems represented by ODE models, and define $\sum ({\textbf {p}})$ as

2.1 $$\begin{aligned} \sum ({\textbf {p}})=\begin{bmatrix} {\textbf {x}}'(t,{\textbf {p}})={\textbf {f}}({\textbf {x}}(t,{\textbf {p}}),{\textbf {p}})+{\textbf {g}}({\textbf {x}}(t,{\textbf {p}}),{\textbf {p}}){\textbf {u}}(t),\\ {\textbf {y}}(t,{\textbf {p}})={\textbf {h}}({\textbf {x}}(t,{\textbf {p}}),{\textbf {p}}), \\ {\textbf {x}}(0,{\textbf {p}})={\textbf {x}}_0({\textbf {p}}), \end{bmatrix} \end{aligned}$$

where ${\textbf {x}}=(x_1,...,x_{n_x})^T\in \mathbb {R}^{n_x}$ is the state vector (the space $\mathbb {R}^{n_x}$ also contains the initial state), ${\textbf {u}}=(u_1,...,u_{n_u})^T\in \mathbb {R}^{n_u}$ is the vector of inputs (which are dependent on the time variable *t*), and ${\textbf {y}}=(y_1,...,y_{n_y})^T\in \mathbb {R}^{n_y}$ is the output vector of experimentally observed quantities. The functions ${\textbf {f}}$ and ${\textbf {g}}$ are analytic functions that describe the evolution of the state variables in time, while ${\textbf {h}}$ determines the model outputs. The vector ${\textbf {p}}\in \Omega \subset \mathbb {R}^{n_p}$, where *Ω * is open and connected, and contains the unknown parameters of the system.

### Definition 2.1

1. A parameter $p_i$, $i=1...n_p$, is *structurally globally (or uniquely) identifiable (s.g.i)* if for almost any $\widetilde{{\textbf {p}}} \in \Omega$,
  $$\begin{aligned} \sum ({\textbf {p}})=\sum (\widetilde{{\textbf {p}}})\Rightarrow p_i=\widetilde{p}_i. \end{aligned}$$
2. A parameter $p_i$, $i=1...n_p$, is *structurally locally identifiable (s.l.i)* if for almost any $\widetilde{{\textbf {p}}} \in \Omega$, there exists a neighborhood ${\textbf {V}}({\textbf {p}})$ of ${\textbf {p}}$ such that
  $$\begin{aligned} \widetilde{{\textbf {p}}}\in {\textbf {V}}({\textbf {p}}) \text { and } \sum ({\textbf {p}})=\sum (\widetilde{{\textbf {p}}})\Rightarrow p_i=\widetilde{p}_i. \end{aligned}$$
3. A parameter $p_i$, $i=1...n_p$, is *structurally unidentifiable* if for almost any $\widetilde{{\textbf {p}}} \in \Omega$, there exists no neighborhood ${\textbf {V}}({\textbf {p}})$ of ${\textbf {p}}$ such that
  $$\begin{aligned} \widetilde{{\textbf {p}}}\in {\textbf {V}}({\textbf {p}}) \text { and } \sum ({\textbf {p}})=\sum (\widetilde{{\textbf {p}}})\Rightarrow p_i=\widetilde{p}_i. \end{aligned}$$

The **model** is then said to be s.l.i if all parameters are s.l.i and s.g.i if all parameters are s.g.i [5].

Next, we introduce several methods to analyse the structural identifiability properties of a model.

### Taylor series method

The Taylor series method for SIA was first developed by Pohjanpalo [25], and can be applied to either linear or nonlinear systems. In [45], we used Taylor Series to analyse linear systems for identifiability properties. Here, we consider nonlinear systems, noting that the algebra involved in applying the method to nonlinear problems can be difficult [4]. We consider a system that is written in the form of Eq. 2.1. Since we assume that the functions ${\textbf {f}}$ and ${\textbf {g}}$ are analytic, they are continuously differentiable. We assume that ${\textbf {h}}$ has infinitely many derivatives with respect to the state variables.

The Taylor series approach exploits the fact that the observations (as given in ${\textbf {y}}(t,{\textbf {p}})$) are unique analytic functions of time and so all their derivatives with respect to time should also be unique, and should, therefore, contain all possible information about the unknown parameters [5, 42]. Thus, the observables can be represented by a Taylor series about the initial state, which can be used to establish identifiability. The Taylor series for ${\textbf {y}}$ in a neighbourhood of the initial state is given by

2.2 $$\begin{aligned} {\textbf {y}}(t,{\textbf {p}}) = {\textbf {y}}(0,{\textbf {p}}) + t \frac{d{\textbf {y}}}{dt}(0,{\textbf {p}}) + \frac{t^2}{2!} \frac{d^{2} {\textbf {y}}}{dt^{2}}(0,{\textbf {p}}) + O(t^3). \end{aligned}$$

We denote

2.3 $$\begin{aligned} {\textbf {a}}_k({\textbf {p}}) = \frac{d^k {\textbf {y}}}{dt^k}(0,{\textbf {p}}),\qquad k=0,1,...,k_{max}, \end{aligned}$$

as the Taylor series derivatives, where $$k_{max}$$ is the maximum number of terms needed for the purpose of determining identifiability properties. Note that the value of $$k_{max}$$ is unknown for nonlinear systems. These coefficients then create a system of nonlinear algebraic equations in the original ODE model parameters. The model is structurally identifiable if $${\textbf {a}}_k({\textbf {p}})={\textbf {a}}_k(\widetilde{{\textbf {p}}})$$ for all $$ k=0,1,...,k_{max}$$ implies that $${\textbf {p}}=\widetilde{{\textbf {p}}}$$ ([5]).

**Fig. 1:**
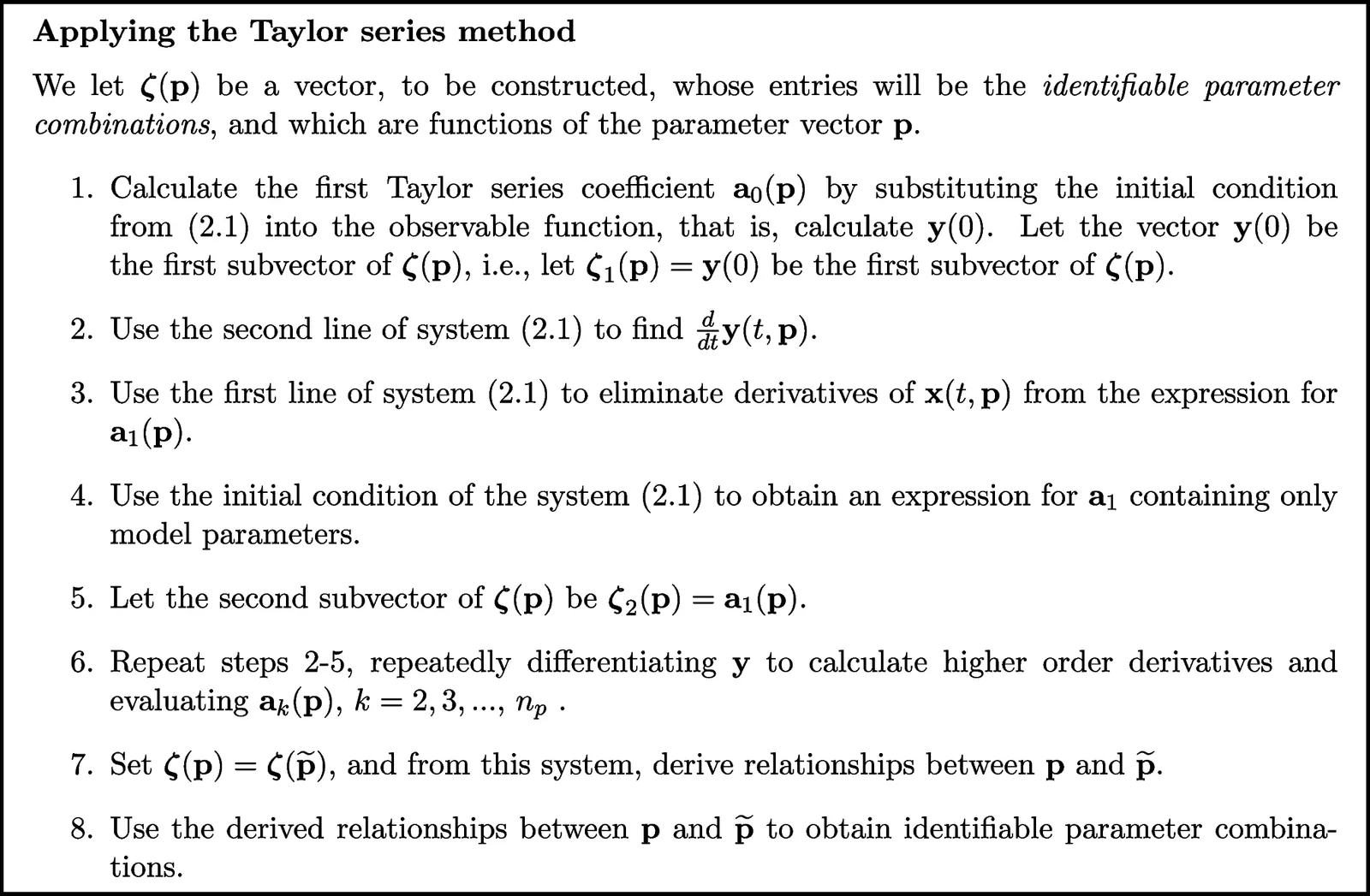
An algorithm for using the Taylor series method to determine identifiabilitynull

For linear problems the number of coefficients needed to determine identifiability is bounded by $k_{max}=2n_x-1$, where $n_x$ is the number of state variables [5]. For nonlinear problems, there is no corresponding a priori bound known for the number of coefficients; this is one of the disadvantages of the Taylor series method for this type of problem. In Fig. 1 we outline the steps needed to use the Taylor series method to determine identifiability of the ODE model.

### Similarity transformation method

The similarity transformation method (or exhaustive modelling approach) was first proposed by Walter and Lecourtier [39], though originally was only applicable to linear problems. This was later extended to include nonlinear problems [35]. Before we discuss the method itself and its implementation, we detail some requirements on the system for the method to be applicable.

#### Controllability and observability

In order to use the similarity transformation method, the system must be a minimal representation, that is it must be both controllable and observable, concepts introduced by Kalman in 1960 [16]. A system is said to be controllable if the system states, in ${\textbf {x}}$, are changed by changing the input, and observable if the initial state ${\textbf {x}}_0$ can be uniquely determined from a set of input-output measurements. For a linear system (unlike Eq. 2.1), in the form

2.4 $$\begin{aligned} \sum ({\textbf {p}})=\begin{bmatrix} {\textbf {x}}'=F{\textbf {x}}+G{\textbf {u}}, \\ {\textbf {y}}=H{\textbf {x}}, \\ {\textbf {x}}(0)={\textbf {x}}_0 \end{bmatrix}, \end{aligned}$$

where *F* is the state matrix, *G* is the state-input matrix and *H* is the state-output matrix, we define the controllability matrix as

2.5 $$\begin{aligned} \mathcal {C}=[G\vdots FG\vdots F^2G\vdots \cdots \vdots F^{n_x-1}G], \end{aligned}$$

and the observability matrix

2.6 $$\begin{aligned} \mathcal {O}=\begin{bmatrix} H \\ HF \\ HF^2 \\ \vdots \\ HF^{n_x-1} \end{bmatrix}, \end{aligned}$$

The system is then said to be controllable if $\text {rank}(\mathcal {C})=n_x$, and observable if $\text {rank}(\mathcal {O})=n_x$ [35]. The underpinning theory, including derivation of these matrices, and the theorems detailing these rank tests can be found in [30].

For a nonlinear system, as in Eq. 2.1, the controllability matrix makes use of the Lie bracket, which is defined as

2.7 $$\begin{aligned} (ad^1_{\textbf {f}},{\textbf {g}})=[{\textbf {f}},{\textbf {g}}]=\frac{\partial {\textbf {g}}({\textbf {x}})}{\partial {\textbf {x}}} {\textbf {f}}({\textbf {x}})-\frac{\partial {\textbf {f}}({\textbf {x}})}{\partial {\textbf {x}}} {\textbf {g}}({\textbf {x}}), \end{aligned}$$

where $(ad^1_{\textbf {f}},{\textbf {g}})$ is the adjoint operator, where the superscript 1 denotes the order of the Lie bracket [41]. Higher order derivatives are defined as

2.8 $$\begin{aligned} (ad^k_{\textbf {f}},{\textbf {g}})&=[f,(ad^{k-1}_{\textbf {f}},{\textbf {g}})]. \end{aligned}$$

The full definition of the Lie bracket can be found in Appendix A, while further explanation of the physical meaning of the Lie bracket and how this relates to controllability can be found in [41]. The controllability matrix is then given by

2.9 $$\begin{aligned} \mathcal {C}=[{\textbf {g}},(ad^1_{\textbf {f}},{\textbf {g}}),\cdots ,(ad^{n-1}_{\textbf {f}},{\textbf {g}})]. \end{aligned}$$

Following this, constructing the observability matrix involves using the *Lie derivative*, which is defined as

2.10 $$\begin{aligned} L^1_{\textbf {f}} {\textbf {y}}({\textbf {x}})=\frac{\partial {\textbf {y}}({\textbf {x}})}{\partial {\textbf {x}}} {\textbf {f}}({\textbf {x}}), \end{aligned}$$

and higher order derivatives can be determined recursively as

2.11 $$\begin{aligned} L^i_{\textbf {f}} {\textbf {y}}(x)=\dfrac{\partial L^{i-1}_{\textbf {f}} {\textbf {y}}(x)}{\partial x} {\textbf {f}}(x). \end{aligned}$$

The full definition of the Lie derivative can be found in Appendix B. The observability matrix is then defined as

2.12 $$\begin{aligned} \mathcal {O}=\begin{bmatrix} \dfrac{\partial L^0_{\textbf {f}} {\textbf {y}}({\textbf {x)}}}{\partial {\textbf {x}}} \\ \dfrac{\partial L^1_{\textbf {f}} {\textbf {y}}({\textbf {x)}}}{\partial {\textbf {x}}} \\ \vdots \\ \dfrac{\partial L^{n-1}_{\textbf {f}} {\textbf {y}}({\textbf {x}})}{\partial {\textbf {x}}} \end{bmatrix}. \end{aligned}$$

Again, the system is controllable and observable if $\text {rank}(\mathcal {C})=n_x$ and $\text {rank}(\mathcal {O})=n_x$.

#### Applying the similarity transformation method

The objective of the similarity transformation method is, given the model Eq. 2.1, with ${\textbf {p}}\in \Omega$, to find all parameter values $\widetilde{{\textbf {p}}}\in \Omega$ and corresponding models of the form

2.13 $$\begin{aligned} \sum (\widetilde{{\textbf {p}}})=\begin{bmatrix} \widetilde{{\textbf {x}}}'(t,\widetilde{{\textbf {p}}})={\textbf {f}}(\widetilde{{\textbf {x}}}(t,\widetilde{{\textbf {p}}}),\widetilde{{\textbf {p}}})+{\textbf {g}}(\widetilde{{\textbf {x}}}(t,\widetilde{{\textbf {p}}}),\widetilde{{\textbf {p}}}){\textbf {u}}(t),\\ {\textbf {y}}(t,\widetilde{{\textbf {p}}})={\textbf {h}}(\widetilde{{\textbf {x}}}(t,\widetilde{{\textbf {p}}}),\widetilde{{\textbf {p}}}), \\ \widetilde{{\textbf {x}}}(0,\widetilde{{\textbf {p}}})=\widetilde{{\textbf {x}}}_0(\widetilde{{\textbf {p}}}). \end{bmatrix} \end{aligned}$$

that have the same input-output map. This involves assuming the existence of an alternate system, $\sum (\widetilde{{\textbf {p}}})$, and applying a set of conditions on this alternate system to ensure that it is equivalent to the original system, $\sum ({\textbf {p}})$. Once equivalence holds, the system is identifiable if

2.14 $$\begin{aligned} \sum ({\textbf {p}})=\sum (\widetilde{{\textbf {p}}})\Rightarrow {\textbf {p}}=\widetilde{{\textbf {p}}}. \end{aligned}$$

#### Linear systems

The linear equivalence of two linear systems is given by the algebraic equivalence theorem [9, 29] for ODE systems. We consider a system in the form of Eq. 2.4, and assume the existence of an alternate system with $\widetilde{F}=F(\widetilde{{\textbf {p}}})$, $\widetilde{G}=G(\widetilde{{\textbf {p}}})$ and $\widetilde{H}=H(\widetilde{{\textbf {p}}})$. These are then equivalent if there exists a matrix $T\in \mathbb {R}^{n_x}\times \mathbb {R}^{n_x}$ such that

2.15a $$\begin{aligned}&\quad \det T \quad \ne 0, \quad \end{aligned}$$

2.15b $$\begin{aligned}&\qquad T\widetilde{{\textbf {x}}}_0 \quad = {\textbf {x}}_0, \quad \end{aligned}$$

2.15c $$\begin{aligned}&\qquad \, T\widetilde{F}\quad =FT, \quad \end{aligned}$$

2.15d $$\begin{aligned}&\qquad \, T\widetilde{G}\quad =G, \quad \end{aligned}$$

2.15e $$\begin{aligned}&\qquad \,\,\, \widetilde{H} \quad =HT,\quad \end{aligned}$$

The full equivalence theorem is given in Appendix C. The process is then to apply the constraints on *F*, *G* and *H* to determine the parameters ${\textbf {p}}$. The system is globally identifiable if it follows from applying these conditions that ${\textbf {p}}=\widetilde{{\textbf {p}}}$ and locally identifiable if there is a finite number of possibilities for each $p_i$.

#### Nonlinear systems

The equivalence theorem has also been extended to include nonlinear systems [4, 5, 35]). With a system in the form of Eq. 2.1, the conditions to ensure equivalence (see Appendix D for the full details) are given by

2.16a $$\begin{aligned} \text {rank}\left( \dfrac{\partial \boldsymbol{\lambda }(\widetilde{{\textbf {x}}})}{\partial \widetilde{{\textbf {x}}}}\right)&= n_x, \quad \end{aligned}$$

2.16b $$\begin{aligned} \quad \boldsymbol{\lambda }(\widetilde{{\textbf {x}}}_0({\textbf {p}}))&= {\textbf {x}}_0(\widetilde{{\textbf {p}}}), \quad \end{aligned}$$

2.16c $$\begin{aligned} \quad f(\boldsymbol{\lambda }(\widetilde{{\textbf {x}}}),{\textbf {p}})&=\dfrac{\partial \boldsymbol{\lambda }(\widetilde{{\textbf {x}}})}{\partial \widetilde{{\textbf {x}}}}f(\widetilde{{\textbf {x}}},\widetilde{{\textbf {p}}}), \quad \end{aligned}$$

2.16d $$\begin{aligned} \quad g(\boldsymbol{\lambda }(\widetilde{{\textbf {x}}}),{\textbf {p}})&=\dfrac{\partial \boldsymbol{\lambda }(\widetilde{{\textbf {x}}})}{\partial \widetilde{{\textbf {x}}}}g(\widetilde{{\textbf {x}}},\widetilde{{\textbf {p}}}), \quad \end{aligned}$$

**Fig. 2:**
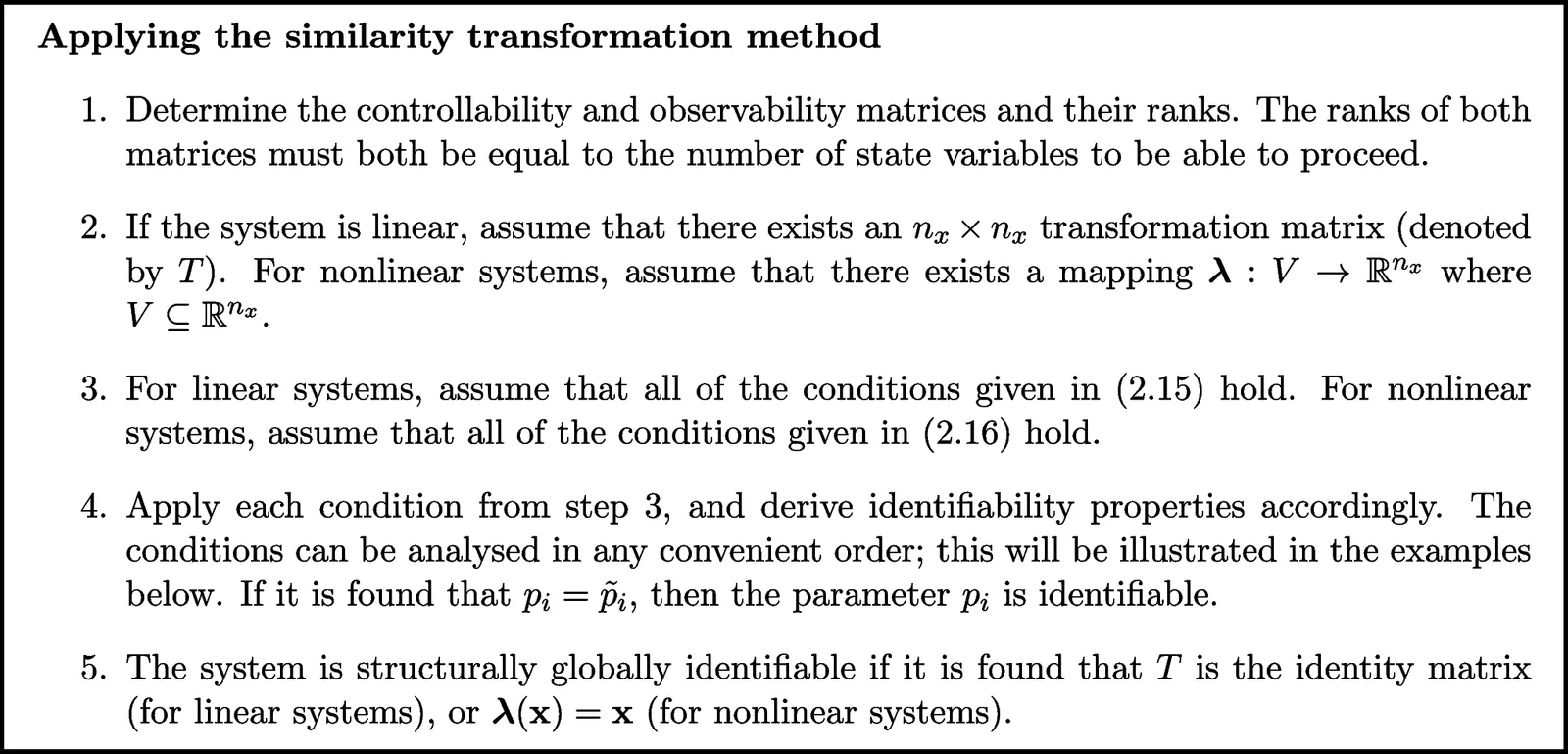
An algorithm for using the similarity transformation method to determine identifiabilitynull

2.16e $$\begin{aligned} \quad h(\boldsymbol{\lambda }(\widetilde{{\textbf {x}}}),{\textbf {p}})&=h(\widetilde{{\textbf {x}}},\widetilde{{\textbf {p}}}), \quad \end{aligned}$$

for all $\widetilde{{\textbf {x}}}\in V$, for some analytical mapping $\boldsymbol{\lambda }(\widetilde{{\textbf {x}}}) \in \mathbb {R}^{n_x}$, where $V\subseteq \mathbb {R}^{n_x}$. A parameter $p_{i}$ is identifiable if it follows from applying these conditions that $p_{i}=\widetilde{p_{i}}$. The system is globally identifiable if it follows from applying these conditions that ${\textbf {p}}=\widetilde{{\textbf {p}}}$ and locally identifiable if there is a finite number of possibilities for each $p_i$. The steps needed to apply this method are summarised in Fig. 2.

With the SIA methods in place, we now proceed to determine the identifiability properties of nonlinear receptor binding models. We begin with an ODE model for ligand binding with ligand depletion at a monomeric receptor.

## Ligand binding monomeric receptor with ligand depletion

In [45], we considered SIA methods applied to linear systems, and applied these to a model of ligand binding to a monomeric receptor. Therein we assumed that the ligand concentration was in excess, that is, the ligand concentration is assumed to be constant throughout the experiment. This assumption is common throughout the literature [17, 18], and yields a linear ODE model for ligand binding [45]. While the constant ligand concentration assumption is valid for many experimental conditions, there are instances where ligand depletion effects may be important [21]. Consideration of ligand depletion effects at a monomeric receptor gives a nonlinear ODE model which serves as a first case study for our nonlinear SIA presentation.

### Model formulation

We consider the binding of a ligand *A* to a receptor *R*, which is represented by the following scheme:where $k_{+}$ and $k_{-}$ are the kinetic association and dissociation constants, respectively. The concentration of ligand *A* is not constant, since it is depleted by the binding reaction. The ODE system that governs the system dynamics is given by

3.1a $$\begin{aligned} \frac{d[A]}{dt}&=k_{-}[AR]- k_{+}[A][R], \end{aligned}$$

3.1b $$\begin{aligned} \frac{d[R]}{dt}&=k_{-}[AR]- k_{+}[A][R], \end{aligned}$$

3.1c $$\begin{aligned} \frac{d[AR]}{dt}&=k_{+}[A][R]- k_{-}[AR], \end{aligned}$$

with initial conditions

3.1d $$\begin{aligned} [A](0)=A_{tot},\qquad [R](0)=R_{tot},\qquad [AR](0)=0. \end{aligned}$$

The signal of interest is the ligand-bound receptor concentration [*AR*]. Experimental data for ligand binding is often obtained using radiolabelled ligands or fluorescence-based methods [11, 33]. Also, we note that kinetic exclusion assay (KinExA) methods offer the possibility of obtaining concentrations as readouts [6]. Here we follow a typical receptor theory analysis approach which assumes that the actual concentration of ligand-bound receptor is the experimental readout [11, 21]. This approach also aligns with the analysis of a pre-formed dimerised receptor binding model [43], whose SIA we consider in Section“Ligand binding pre-dimerised receptor with ligand depletion”. In Section“Ligand-induced dimerisation (LID)”, as an alternative approach, we extend the SIA to account for the case where the measured readout is proportional to ligand-bound receptor concentration, as considered in the ligand-induced dimerisation model in [44].

We note that both the total receptor concentration and the total ligand concentration are conserved, so that

3.2a $$\begin{aligned} [A]+[AR]&= A_{tot}, \end{aligned}$$

3.2b $$\begin{aligned} [R]+[AR]&= R_{tot}. \end{aligned}$$

Using these relations, the ODE system Eq. 3.1 reduces to the scalar problem

3.3a $$\begin{aligned} \frac{d[AR]}{dt} = k_{+}[AR]^{2} - b[AR] + c, \qquad [AR](0)=0, \end{aligned}$$

where

3.3b $$\begin{aligned} b = k_{+}(A_{tot}+R_{tot}) + k_{-}, \qquad c = k_{+}A_{tot}R_{tot}. \end{aligned}$$

### Exact solution

Typically, nonlinear receptor theory models do not yield exact solutions (for example, the ligand-induced dimerisation model in Section“Ligand-induced dimerisation (LID)”). It is worth noting here that the initial value problem Eq. 3.3 may be solved exactly by separation of variables, giving

3.4 $$\begin{aligned} [AR](t) = \frac{X_{+}X_{-}\left( 1-e^{-\sqrt{b^{2}-4k_{+}c}\; {t}}\right) }{X_{+}-X_{-}e^{-\sqrt{b^{2}-4k_{+}c}\; {t}}}, \end{aligned}$$

where

3.5 $$\begin{aligned} X_{\pm } = \frac{b\pm \sqrt{b^{2}-4k_{+}c}}{2k_{+}}. \end{aligned}$$

### SIA for ligand depletion model - Taylor series

The initial value problem describing the kinetics of the system is given in Eq. 3.1, where $R_{tot}$ is the total receptor concentration and $A_{tot}$ the total concentration of ligand. The concentration of *[AR]* is measured experimentally, hence the observed output is

3.6 $$\begin{aligned} y=[AR]. \end{aligned}$$

The total ligand concentration $A_{tot}$ is known, so we have the unknown parameter vector ${\textbf {p}}=(k_{+},k_{-},R_{tot})^{T}$.

The Taylor series of *y* about *t=0*, noting that we use the bracketed superscript to indicate the order of the derivative, is

3.7 $$\begin{aligned} y(t)=y(0)+ty^{(1)}(0)+\frac{t^2}{2!}y^{(2)}(0)+... \end{aligned}$$

Since, we have $t_0=0$, the first coefficient is simply

3.8 $$\begin{aligned} a_{0} = y(0). \end{aligned}$$

As *y=[AR]*, we substitute initial conditions into the equation to obtain the first coefficient as

3.9 $$\begin{aligned} a_{0}=y(0)=[AR](0)=0. \end{aligned}$$

This gives no information about parameter identifiability as the expression for $a_{0}$ contains no parameters.

We find the next coefficient by calculating the first derivative of the output function, namely

3.10 $$\begin{aligned} y^{(1)}(t)=[AR]^{(1)}(t). \end{aligned}$$

Substituting the ODE for *[AR]* in Eq. 3.1 gives

3.11 $$\begin{aligned} y^{(1)}=k_{+}[A][R]-k_{-}[AR]. \end{aligned}$$

Evaluating at *t=0*, and then using the initial conditions, gives the second coefficient as

3.12 $$\begin{aligned} a_{1} = y^{(1)}(0)=k_{+}A_{tot}R_{tot}. \end{aligned}$$

As $A_{tot}$ is known this can be simplified to give

3.13 $$\begin{aligned} c_1=k_{+}R_{tot}, \end{aligned}$$

where we denote $c_i$ as the identifiable parameter combination. Further derivatives are calculated with recursive substitution of the equations in system Eq. 3.1. The second derivative is found to be given by

3.14 $$\begin{aligned} y^{(2)}=[AR]^{(2)}&= k_{+}\Big ( [A]^{(1)}[R]+ [A][R]^{(1)} \Big ) -k_{-}[AR]^{(1)} \nonumber \\&= k_{+}\Big ([A]+[R]\Big ) \, \Big (k_{-}[AR]- k_{+}[A][R]\Big )\\& -k_{-}\Big (k_{+}[A][R]- k_{-}[AR]\Big ) \nonumber \\&= \Big (k_{+}[R]+ k_{+}[A]+k_{-}\Big ) \, \Big (k_{-}[AR]- k_{+}[A][R]\Big ). \end{aligned}$$

Applying the initial conditions, we find that

3.15 $$\begin{aligned} y^{(2)}(0)=-k_{+}A_{tot}R_{tot}\big (k_{+}R_{tot}+k_{+}A_{tot}+k_{-}\big ). \end{aligned}$$

We note that

3.16 $$\begin{aligned} y^{(2)}(0)=-c_1A_{tot}\big (c_{1}+k_{+}A_{tot}+k_{-}\big ), \end{aligned}$$

and since $A_{tot}$ and $c_1$ are known, we can write our second identifiable parameter combination as

3.17 $$\begin{aligned} c_2=k_{+}A_{tot}+k_{-}. \end{aligned}$$

Continuing this process, we find that

3.18 $$\begin{aligned} y^{(3)}(0)&=k_{+}A_{tot}R_{tot}(k_{+}^2A_{tot}^2 + 4k_{+}^2A_{tot}R_{tot}+ k_{+}^2R_{tot}^2 \\&+ 2k_{+}k_{-}A_{tot}+ 2k_{+}k_{-}R_{tot}+ k_{-}^2). \end{aligned}$$

This can be written, using coefficients $c_1$ and $c_2$, as

3.19 $$\begin{aligned} y^{(3)}(0)= c_1A_{tot}\Big (2k_{+}c_1A_{tot}+(c_{1}+c_{2})^2\Big ). \end{aligned}$$

As $c_1$, $c_2$ and $A_{tot}$ are known, we have

3.20 $$\begin{aligned} c_3 = k_{+}, \end{aligned}$$

that is, the parameter $k_{+}$ is identifiable. With $k_{+}$ known, we can find $R_{tot}$ from $c_1$, as

3.21 $$\begin{aligned} R_{tot}= \frac{c_1}{k_{+}}. \end{aligned}$$

Finally, with $k_{+}$, $A_{tot}$ and $R_{tot}$ known, we can find $k_{-}$ from identifiable combination $c_2$. Hence, we find all parameters to be identifiable, and thus the system is globally structurally identifiable. This result is in clear contrast to the non-identifiable single-timecourse scenario for the corresponding model without ligand depletion [45]. A somewhat intuitive explanation for this comes from the fact that ligand depletion results in different values of available ligand concentration as the system evolves, and using a range of ligand concentrations to generate multiple timecourses is known to yield an identifiable model for the no-depletion case [45].

### SIA for ligand depletion model - similarity transformation

We have established the identifiability properties of the system Eq. 3.1 (and, equivalently, the scalar problem Eq. 3.3) using the Taylor Series approach. We now illustrate the derivation of the same results using the similarity transformation method presented in Section“Similarity transformation method”.

We can write the scalar problem Eq. 3.3 in terms of the notation used in Eq. 2.1 by considering a scalar input *u=1* together with the scalar quantities

3.22a $$\begin{aligned} x&= [AR], \end{aligned}$$

3.22b $$\begin{aligned} f(x(t,{\textbf {p}}))&= k_{+}x^{2}-\big (k_{+}(A_{tot}+R_{tot}) + k_{-}\big )x, \end{aligned}$$

3.22c $$\begin{aligned} g(x(t,{\textbf {p}}))&= k_{+}A_{tot}R_{tot}, \end{aligned}$$

3.22d $$\begin{aligned} h(x(t,{\textbf {p}}),{\textbf {p}})&= x, \end{aligned}$$

3.22e $$\begin{aligned} x_0({\textbf {p}})&= 0. \end{aligned}$$

We remark that *g* is nonzero so that the controllability matrix is nonzero. The concentration *[AR]* is measured experimentally, hence the observed output is

3.22f $$\begin{aligned} y=[AR]. \end{aligned}$$

We first note that the model is observable and identifiable, since it is a single ODE.

Next, we assume that there exists a transformation *λ (x)* that satisfies all of the conditions in Eq. 2.16. First, condition Eq. 2.16e gives

3.23 $$\begin{aligned} \lambda ([AR])=[AR]. \end{aligned}$$

Considering point 5 of the method outlined in Fig. 2, we can now conclude that the system is structurally globally identifiable, agreeing with the result of Section“SIA for ligand depletion model - Taylor series”. For completeness and illustration, however, we now present the calculations which derive individual parameter identifiability results, using point 3 from Fig. 2.

Since *λ (x)=x*, conditions Eqs. 2.16a and 2.16b hold true. Differentiating, we have

3.24 $$\begin{aligned} \frac{d\lambda }{dx}=1. \end{aligned}$$

Checking condition Eq. 2.16d gives

3.25 $$\begin{aligned} k_{+}A_{tot}R_{tot}= \widetilde{k_{+}}A_{tot}\widetilde{R_{tot}}\qquad \Rightarrow \qquad k_{+}R_{tot}= \widetilde{k_{+}}\widetilde{R_{tot}}. \end{aligned}$$

Setting condition Eq. 2.16c gives that

3.26 $$\begin{aligned}& k_{+}[AR]^2-(k_{+}A_{tot}+k_{+}R_{tot}+k_{-})[AR]\\&= \widetilde{k_{+}}[AR]^2-(\widetilde{k_{+}}A_{tot}+\widetilde{k_{+}}\widetilde{R_{tot}}+\widetilde{k_{-}})[AR]. \end{aligned}$$

Equating coefficients of *[AR]* then gives

3.27 $$\begin{aligned} k_{+}&= \widetilde{k_{+}}, \end{aligned}$$

3.28 $$\begin{aligned} k_{+}A_{tot}+k_{+}R_{tot}+k_{-}&= \widetilde{k_{+}}A_{tot}+\widetilde{k_{+}}\widetilde{R_{tot}}+\widetilde{k_{-}}. \end{aligned}$$

Taking these together with Eq. 3.25 we find that all parameters are identifiable, and as such, the system itself is globally structurally identifiable.

## Ligand binding pre-dimerised receptor with ligand depletion

The dimer model for constant concentration ligand binding introduced in [43] is non-identifable when considering a single timecourse output [45]. We now consider whether modifying this to give a corresponding ligand depletion model results in global identifiability as it did for the monomer case.

### Model formulation

The GPCR homodimer model presented and analysed in [43], for a single ligand *A*, considers the following schematic:Here, *R* represents dimerised receptor, *AR* represents dimerised receptor with one protomer of the dimer bound by ligand, and *ARA* represents dimerised receptor with both protomers bound by ligand. The parameters $k_{+}$ and $k_{-}$ are the binding and dissociation rate constants respectively, and the parameters $\alpha _{+}$ and $\alpha _{-}$ are the forward and backward binding cooperativities respectively.

The ODE system describing the model dynamics is given as

4.1a $$\begin{aligned} \frac{d[A]}{dt}&=-k_{+}[A][R]+k_{-}[AR]- \alpha _+k_{+}[A][AR]+ \alpha _-k_{-}[ARA], \end{aligned}$$

4.1b $$\begin{aligned} \frac{d[R]}{dt}&=-k_{+}[A][R]+k_{-}[AR], \end{aligned}$$

4.1c $$\begin{aligned} \frac{d[AR]}{dt}&= k_{+}[A][R]-(k_{-}+\alpha _+k_{+}[A])[AR]+\alpha _-k_{-}[ARA], \end{aligned}$$

4.1d $$\begin{aligned} \frac{d[ARA]}{dt}&=\alpha _+k_{+}[A][AR]-\alpha _-k_{-}[ARA], \end{aligned}$$

with initial conditions

4.1e $$\begin{aligned} [A](0)=A_{tot}, \qquad [R](0)=R_{tot},\qquad [AR](0)=0,\qquad [ARA](0)=0, \end{aligned}$$

where $R_{tot}$ is the total receptor concentration and $A_{tot}$ is the initial ligand concentration. The measured quantity is bound ligand, and the output *y* is given by

4.1f $$\begin{aligned} y=[AR]+2[ARA]. \end{aligned}$$

We assume the only known parameter is the initial ligand concentration, $A_{tot}$, and so we have the vector of unknown parameters as ${\textbf {p}}=(\alpha _+,\alpha _-,k_{+},k_{-},R_{tot})$.

Again, the concentrations of receptors and ligand are conserved, so we have

4.2 $$\begin{aligned} R_{tot}=[R]+[AR]+[ARA], \qquad A_{tot}= [A]+ [AR]+ 2[ARA]. \end{aligned}$$

We use these conservation laws to reduce the system to the following two-component system:

4.3a $$\begin{aligned} \frac{d[AR]}{dt}&= (k_{+}+ \alpha _+k_{+})[AR]^2 + (3k_{+}+ 2\alpha _+k_{+})[AR][ARA]\\&-(k_{+}A_{tot}+ k_{+}R_{tot}+ \alpha _+k_{+}A_{tot}+ k_{-})[AR]\\&+ 2k_{+}[ARA]^2 + (\alpha _-k_{-}- k_{+}A_{tot}- 2k_{+}R_{tot})[ARA]\\&+ k_{+}A_{tot}R_{tot}, \end{aligned}$$

4.3b $$\begin{aligned} \frac{d[ARA]}{dt}&= -\alpha _+k_{+}[AR]^2 - 2\alpha _+k_{+}[AR][ARA]\\&+ \alpha _+k_{+}A_{tot}[AR]- \alpha _-k_{-}[ARA]. \end{aligned}$$

In Section“SIA for ligand depletion dimer model - Taylor series”, we apply the Taylor Series approach to the system Eq. 4.1, and in Section“SIA for ligand depletion dimer model - similarity transformation”, we apply the similarity transformation approach to Eq. 4.3 since controllability is easily verified for the lower dimensional system.

### SIA for ligand depletion dimer model - Taylor series

In this section, we apply the Taylor Series approach to the system Eq. 4.1, The Taylor series for ${\textbf {y}}$ about *t=0* is

4.4 $$\begin{aligned} y(t)=y(0)+ty^{(1)}(0)+\frac{t^2}{2!}y^{(2)}(0)+... \; , \end{aligned}$$

where bracketed superscript is used to indicate the order of the derivative. The first coefficient is simply

4.5 $$\begin{aligned} a_{0} = y(0)=0. \end{aligned}$$

From Eq. 4.1, we find that the first derivative of the output function is given by

4.6 $$\begin{aligned} y^{(1)}&=k_{+}[A][R]-(k_{-}+\alpha _+k_{+}[A])[AR]+\alpha _-k_{-}[ARA]\\&+ 2(\alpha _+k_{+}[A][AR]-\alpha _-k_{-}[ARA])= k_{+}[A][R]-(k_{-} \nonumber \\&-\alpha _+k_{+}[A])[AR]-\alpha _-k_{-}[ARA], \end{aligned}$$

giving the unique coefficient corresponding to the first derivative as

4.7 $$\begin{aligned} y^{(1)}(0)=k_{+}A_{tot}R_{tot}. \end{aligned}$$

The remaining coefficients are determined using MATLAB’s Symbolic Toolbox [34], giving

4.8 $$\begin{aligned} y^{(2)}(0)&=-k_{+}A_{tot}R_{tot}\big (k_{-}+ k_{+}A_{tot}+ k_{+}R_{tot}- \alpha _+k_{+}A_{tot}\big ) \end{aligned}$$

4.9 $$\begin{aligned} y^{(3)}(0)&= k_{+}A_{tot}R_{tot}\big (- \alpha _+^2k_{+}^2A_{tot}^2 - \alpha _+k_{+}^2A_{tot}^2 - 4\alpha _+k_{+}^2A_{tot}R_{tot}\\&- \alpha _-\alpha _+k_{+}k_{-}A_{tot}+ k_{+}^2A_{tot}^2+ 4k_{+}^2A_{tot}R_{tot}+ k_{+}^2R_{tot}^2 \nonumber \\& + 2k_{+}k_{-}A_{tot}+ 2k_{+}k_{-}R_{tot}+ k_{-}^2\big ), \end{aligned}$$

4.10 $$\begin{aligned} y^{(4)}(0)&= k_{+}A_{tot}R_{tot}\big (\alpha _+^3k_{+}^3A_{tot}^3 + 2\alpha _+^2\alpha _-k_{+}^2k_{-}A_{tot}^2 + \alpha _+^2k_{+}^3A_{tot}^3\nonumber \\&\quad + 3\alpha _+^2k_{+}^3A_{tot}^2R_{tot}+ \alpha _+^2k_{+}^2k_{-}A_{tot}^2 + \alpha _+\alpha _-^2k_{+}k_{-}^2A_{tot}\nonumber \\&\quad+ \alpha _+\alpha _-k_{+}^2k_{-}A_{tot}^2 + 4\alpha _+\alpha _-k_{+}^2k_{-}A_{tot}R_{tot}+ \alpha _+k_{+}^3A_{tot}^3 \nonumber \\&\quad + 15\alpha _+k_{+}^3A_{tot}^2R_{tot}+ 11\alpha _+k_{+}^3A_{tot}R_{tot}^2 + 6\alpha _+k_{+}^2k_{-}A_{tot}R_{tot}\nonumber \\&\quad- \alpha _+k_{+}k_{-}^2A_{tot}- k_{+}^3A_{tot}^3 - 11k_{+}^3A_{tot}^2R_{tot}- 11k_{+}^3A_{tot}R_{tot}^2\nonumber \\&\quad - k_{+}^3R_{tot}^3 - 3k_{+}^2k_{-}A_{tot}^2 - 14k_{+}^2k_{-}A_{tot}R_{tot}- 3k_{+}^2k_{-}R_{tot}^2 \nonumber \\&\quad - 3k_{+}k_{-}^2A_{tot}- 3k_{+}k_{-}^2R_{tot}- k_{-}^3\big ), \end{aligned}$$

4.11 $$\begin{aligned} y^{(5)}(0)&= -k_{+}A_{tot}R_{tot}\big (\alpha _+^4k_{+}^4A_{tot}^4 + 3\alpha _+^3\alpha _-k_{+}^3k_{-}A_{tot}^3 + \alpha _+^3k_{+}^4A_{tot}^4 \nonumber \\&\quad- 4\alpha _+^3k_{+}^4A_{tot}^3R_{tot}+ 2\alpha _+^3k_{+}^3k_{-}A_{tot}^3 + 3\alpha _+^2\alpha _-^2k_{+}^2k_{-}^2A_{tot}^2\nonumber \\&\quad + 2\alpha _+^2\alpha _-k_{+}^3k_{-}A_{tot}^3 + 6\alpha _+^2\alpha _-k_{+}^3k_{-}A_{tot}^2R_{tot}+ 2\alpha _+^2\alpha _-k_{+}^2k_{-}^2A_{tot}^2\nonumber \\&\quad + \alpha _+^2k_{+}^4A_{tot}^4 + 11\alpha _+^2k_{+}^4A_{tot}^3R_{tot}- 2\alpha _+^2k_{+}^4A_{tot}^2R_{tot}^2\nonumber \\&\quad + 2\alpha _+^2k_{+}^3k_{-}A_{tot}^3 + 18\alpha _+^2k_{+}^3k_{-}A_{tot}^2R_{tot} + \alpha _+\alpha _-^3k_{+}k_{-}^3A_{tot}+\nonumber \\&\quad \alpha _+\alpha _-^2k_{+}^2k_{-}^2A_{tot}^2 + 4\alpha _+\alpha _-^2k_{+}^2k_{-}^2A_{tot}R_{tot}+ \alpha _+\alpha _-k_{+}^3k_{-}A_{tot}^3\nonumber \\&\quad + 16\alpha _+\alpha _-k_{+}^3k_{-}A_{tot}^2R_{tot}+ 11\alpha _+\alpha _-k_{+}^3k_{-}A_{tot}R_{tot}^2 + \nonumber \\&\quad6\alpha _+\alpha _-k_{+}^2k_{-}^2A_{tot}R_{tot}- \alpha _+\alpha _-k_{+}k_{-}^3A_{tot} + \alpha _+k_{+}^4A_{tot}^4 \nonumber \\&\quad+ 41\alpha _+k_{+}^4A_{tot}^3R_{tot}+ 100\alpha _+k_{+}^4A_{tot}^2R_{tot}^2 + 26\alpha _+k_{+}^4A_{tot}R_{tot}^3 \nonumber \\&\quad+ 37\alpha _+k_{+}^3k_{-}A_{tot}^2R_{tot} + 34\alpha _+k_{+}^3k_{-}A_{tot}R_{tot}^2 - 3\alpha _+k_{+}^2k_{-}^2A_{tot}^2\nonumber \\&\quad + 6\alpha _+k_{+}^2k_{-}^2A_{tot}R_{tot}- 2\alpha _+k_{+}k_{-}^3A_{tot}- k_{+}^4A_{tot}^4 \nonumber \\&\quad - 26k_{+}^4A_{tot}^3R_{tot}- 66k_{+}^4A_{tot}^2R_{tot}^2 - 26k_{+}^4A_{tot}R_{tot}^3 - k_{+}^4R_{tot}^4 \nonumber \\&\quad- 4k_{+}^3k_{-}A_{tot}^3 - 56k_{+}^3k_{-}A_{tot}^2R_{tot}- 56k_{+}^3k_{-}A_{tot}R_{tot}^2 \nonumber \\&\quad- 4k_{+}^3k_{-}R_{tot}^3 - 6k_{+}^2k_{-}^2A_{tot}^2 - 34k_{+}^2k_{-}^2A_{tot}R_{tot}\nonumber \\&\quad - 6k_{+}^2k_{-}^2R_{tot}^2 - 4k_{+}k_{-}^3A_{tot}- 4k_{+}k_{-}^3R_{tot}- k_{-}^4\big ). \end{aligned}$$

There is no systematic method for reducing the set of unwieldy coefficients down to identifiable combinations. Therefore, we conclude that the Taylor Series approach for this dimerised receptor model appears to be intractable. An ad hoc approach may yield identifiable combinations, but we do not pursue this further. At this point, we abandon the Taylor Series approach and continue our SIA by implementing the similarity transformation method.

### SIA for ligand depletion dimer model - similarity transformation

In reduced form the governing system for the dimerised receptor model is

4.12a $$\begin{aligned} \frac{d[AR]}{dt}&= (k_{+}+ \alpha _+k_{+})[AR]^2 + (3k_{+}+ 2\alpha _+k_{+})[AR][ARA]\nonumber \\&\qquad -(k_{+}A_{tot}+ k_{+}R_{tot}+ \alpha _+k_{+}A_{tot}+ k_{-})[AR]\nonumber \\&\qquad + 2k_{+}[ARA]^2 + (\alpha _-k_{-}- k_{+}A_{tot}- 2k_{+}R_{tot})[ARA]+ k_{+}A_{tot}R_{tot}, \end{aligned}$$

4.12b $$\begin{aligned} \frac{d[ARA]}{dt}&= -\alpha _+k_{+}[AR]^2 - 2\alpha _+k_{+}[AR][ARA]+ \alpha _+k_{+}A_{tot}[AR]\\&- \alpha _-k_{-}[ARA], \end{aligned}$$

with initial conditions

4.12c $$\begin{aligned} [AR](0)=0,\qquad [ARA](0)=0, \end{aligned}$$

We can write the problem Eq. 4.12 in terms of the notation used in Eq. 2.1 by considering an input ${\textbf {u}}=[1]$ together with the quantities

4.13a $$\begin{aligned} \textbf{f}&= \begin{bmatrix} \begin{aligned} & \Big \{k_{+}(1 + \alpha _+)[AR]^2 + 2k_{+}[ARA]^2 + k_{+}(3 + 2\alpha _+)[AR][ARA]\Big . \\ & \; \Big . -\Big (k_{+}(1+\alpha _+)A_{tot}+ k_{+}R_{tot}+ k_{-}\Big )[AR]+ (\alpha _-k_{-}- k_{+}A_{tot}- 2k_{+}R_{tot})[ARA]\Big \} \end{aligned} -\alpha _+k_{+}[AR]^2 - 2\alpha _+k_{+}[AR][ARA]+ \alpha _+k_{+}A_{tot}[AR]- \alpha _-k_{-}[ARA]\end{bmatrix}, \end{aligned}$$

4.13b $$\begin{aligned} \textbf{g}&= \begin{bmatrix} k_{+}A_{tot}R_{tot}\\ 0 \end{bmatrix}, \end{aligned}$$

4.13c $$\begin{aligned} y&= [AR]+2[ARA], \end{aligned}$$

4.13d $$\begin{aligned} h&= [1 \;\; 2]\textbf{x}, \end{aligned}$$

4.13e $$\begin{aligned} \textbf{x}&= \begin{bmatrix} [AR] \\ [ARA] \end{bmatrix},\end{aligned}$$

4.13f $$\begin{aligned} \textbf{x}_{0}&= \begin{bmatrix} 0 \\ 0 \end{bmatrix}, \end{aligned}$$

where $n_{x}=2$. We now apply the similarity transformation method by following the steps outlined in Fig. 2.

#### Observability and controllability rank conditions

First, we check the observability rank condition by making use of the Lie derivative and observability matrix that were defined in Eqs. 2.9-2.10. For $n_{x}=2$, we construct the observability matrix $\mathcal {O}$, given by

4.14 $$\begin{aligned} \mathcal {O}=\begin{bmatrix} \dfrac{\partial L^0_{\textbf {f}}y({\textbf {x)}}}{\partial {\textbf {x}}} \\ \dfrac{\partial L^1_{\textbf {f}}y({\textbf {x)}}}{\partial {\textbf {x}}} \end{bmatrix}. \end{aligned}$$

Recall that

4.15 $$\begin{aligned} L_{\textbf {f}}^0 y({\textbf {x}})=y({\textbf {x}}), \qquad L^1_{\textbf {f}}y({\textbf {x}})=\frac{\partial y({\textbf {x}})}{\partial {\textbf {x}}} {\textbf {f}}({\textbf {x}}). \end{aligned}$$

For our model, we have

4.16 $$\begin{aligned} L_{\textbf {f}}^0 y({\textbf {x}}) = [1 \;\; 2]\textbf{x}, \;\; \Longrightarrow \;\; \dfrac{\partial L^0_{\textbf {f}}y({\textbf {x)}}}{\partial {\textbf {x}}} = [1 \;\; 2]. \end{aligned}$$

Also, we have

4.17 $$\begin{aligned} L_{\textbf {f}}^1 y({\textbf {x}})&= \frac{\partial y({\textbf {x}})}{\partial {\textbf {x}}} {\textbf {f}}({\textbf {x}}) \nonumber \\&= k_{+}(1-\alpha _+)[AR]^2 + k_{+}(3-2\alpha _+)[AR][ARA]\nonumber \\&\qquad+ (\alpha _+k_{+}A_{tot}- k_{+}A_{tot}- k_{+}R_{tot}- k_{-})[AR]\nonumber \\&\qquad + 2k_{+}[ARA]^2 - (k_{+}A_{tot}+ 2k_{+}R_{tot}+ \alpha _-k_{-})[ARA], \end{aligned}$$

which gives

4.18 $$\begin{aligned} \dfrac{\partial L^1_{\textbf {f}}y({\textbf {x)}}}{\partial {\textbf {x}}} = \begin{bmatrix} \begin{aligned} & \Big \{ 2k_{+}(1-\alpha _+)[AR]+ k_{+}(3-2\alpha _+)[ARA]\qquad \Big .\\ & \Big . + (\alpha _+k_{+}A_{tot}- k_{+}A_{tot}- k_{+}R_{tot}- k_{-}) \Big \}\end{aligned} & \begin{aligned} & \Big \{k_{+}(3-2\alpha _+)[AR]+ 4k_{+}[ARA]\Big .\\ & \Big . - (k_{+}A_{tot}+ 2k_{+}R_{tot}+ \alpha _-k_{-}) \Big \} \end{aligned} \end{bmatrix} . \end{aligned}$$

This results in the following observability matrix:

4.19 $$\begin{aligned} \mathcal {O}=\begin{bmatrix} 1 & 2 \\ \begin{aligned} & \Big \{ 2k_{+}(1-\alpha _+)[AR]+ k_{+}(3-2\alpha _+)[ARA]\qquad \Big .\\ [-0.3ex] & \Big . \;\; + (\alpha _+k_{+}A_{tot}- k_{+}A_{tot}- k_{+}R_{tot}- k_{-}) \Big \}\end{aligned} & \begin{aligned} & \Big \{k_{+}(3-2\alpha _+)[AR]+ 4k_{+}[ARA]\Big .\\ [-0.3ex] & \Big . \;\; - (k_{+}A_{tot}+ 2k_{+}R_{tot}+ \alpha _-k_{-}) \Big \} \end{aligned} \end{bmatrix} . \end{aligned}$$

We note that $\mathcal {O}$ has $\textrm{rank}(\mathcal {O})=n_{x}=2$, so the observability rank criterion is satisfied.

Next, we determine the controllability matrix using the Lie bracket, which was defined in Eq. 2.7. For $n_{x}=2$, we construct the observability matrix $\mathcal {C}$, given by

4.20 $$\begin{aligned} \mathcal {C}=[{\textbf {g}},(ad^1_{\textbf {f}},{\textbf {g}})], \end{aligned}$$

where we recall that

$$\begin{aligned} (ad^1_{\textbf {f}},{\textbf {g}})=[{\textbf {f}},{\textbf {g}}]=\frac{\partial {\textbf {g}}({\textbf {x}})}{\partial {\textbf {x}}} {\textbf {f}}({\textbf {x}})-\frac{\partial {\textbf {f}}({\textbf {x}})}{\partial {\textbf {x}}} {\textbf {g}}({\textbf {x}}). \end{aligned}$$

For our model, $\textbf{f}$ is given by Eq. 4.13a and $\textbf{g}$ is given by Eq. 4.13b, so that

$$\begin{aligned} \frac{\partial {\textbf {g}}({\textbf {x}})}{\partial {\textbf {x}}} = \begin{bmatrix} 0 & 0 \\ 0 & 0 \end{bmatrix}, \end{aligned}$$

and

$$\begin{aligned} \frac{\partial {\textbf {f}}({\textbf {x}})}{\partial {\textbf {x}}} = \begin{bmatrix} J_{11} & J_{12} \\ J_{21} & J_{22} \end{bmatrix}, \end{aligned}$$

where

$$\begin{aligned} J_{11}&= 2k_{+}(1+\alpha _{+})[AR] - ((1+\alpha _{+})k_{+}A_{tot}+k_{+}R_{tot}+k_{-}) + k_{+}(3+2\alpha _{+})[ARA], \\ J_{12}&= k_{+}(3+2\alpha _{+})[AR] + 4k_{+}[ARA] + (\alpha _{-}k_{-}-k_{+}A_{tot}-2k_{+}R_{tot}), \\ J_{21}&=\alpha _{+}k_{+}\big (A_{tot}-2[AR]-2[ARA]\big ), \\ J_{22}&= - \big (2\alpha _{+}k_{+}[AR]+\alpha _{-}k_{-}\big ). \end{aligned}$$

Therefore the controllability matrix $\mathcal {C}$ is given by

4.21 $$\begin{aligned} \mathcal {C}= k_{+}A_{tot}R_{tot}\begin{bmatrix} 1 & -J_{11} \\ 0 & -J_{21} \end{bmatrix}, \end{aligned}$$

which has $\textrm{rank}(\mathcal {C})=n_{x} = 2$, so the controllability rank criterion is satisfied. Thus the system is both observable and controllable, and we proceed with the similarity transformation method.

#### Applying the method

All conditions in Eq. 2.16 should be satisfied, and we can consider them simultaneously. Here, we subsequently assume them to hold in an order that gives the most immediate results. We consider a transformation $\boldsymbol{\lambda }({\textbf {x}})={[}\lambda _1({\textbf {x}}),\lambda_{2} ({\textbf {x}}){]}^{T}$, and we first assume that condition Eq. 2.16e holds, such that ${\textbf {h}}(\boldsymbol{\lambda }({\textbf {x}}),{\textbf {p}})={\textbf {h}}({\textbf {x}},\widetilde{{\textbf {p}}})$. This gives

4.22 $$\begin{aligned} \lambda _1+2\lambda_{2} =[AR]+2[ARA],\qquad \Longrightarrow \qquad \lambda _1=[AR]+2[ARA]-2\lambda_{2}. \end{aligned}$$

Next, note that Eqs. 2.16a, 2.16c and 2.16d all involve the Jacobian

$$\begin{aligned} \displaystyle {\frac{\partial \boldsymbol{\lambda }}{\partial {\textbf {x}}} = \begin{bmatrix} \frac{\partial \lambda _1}{\partial [AR]} & \frac{\partial \lambda _1}{\partial [ARA]} \\ \frac{\partial \lambda_2 }{\partial [AR]} & \frac{\partial \lambda_2 }{\partial [ARA]} \end{bmatrix}.} \end{aligned}$$

Differentiating Eq. 4.22 with respect to *[AR]* gives

4.23 $$\begin{aligned} \frac{\partial \lambda _1}{\partial [AR]}=1-2\frac{\partial \lambda_2 }{\partial [AR]}. \end{aligned}$$

Similarly, differentiating Eq. 4.22 with respect to *[ARA]* gives

4.24 $$\begin{aligned} \frac{\partial \lambda _1}{\partial [ARA]}=2-2\frac{\partial \lambda_2 }{\partial [ARA]}. \end{aligned}$$

Hence the Jacobian of $\boldsymbol{\lambda }({\textbf {x}})$ is given by

4.25 $$\begin{aligned} \frac{\partial \boldsymbol{\lambda }}{\partial {\textbf {x}}}=\begin{bmatrix} 1-2\dfrac{\partial \lambda_2 }{\partial [AR]} & 2-2\dfrac{\partial \lambda_2 }{\partial [ARA]} \\ \dfrac{\partial \lambda_2 }{\partial [AR]} & \dfrac{\partial \lambda_2 }{\partial [ARA]} \end{bmatrix}. \end{aligned}$$

We next consider condition Eq. 2.16d, namely

$$\begin{aligned} {\textbf {g}}(\boldsymbol{\lambda }({\textbf {x}}),{\textbf {p}})=\dfrac{\partial \boldsymbol{\lambda }({\textbf {x}})}{\partial {\textbf {x}}} {\textbf {g}}({\textbf {x}},\widetilde{{\textbf {p}}}). \end{aligned}$$

For our system, this gives

4.26 $$\begin{aligned} \begin{bmatrix}k_{+}A_{tot}R_{tot}\\ 0 \end{bmatrix}&= \begin{bmatrix} 1-2\dfrac{\partial \lambda_2 }{\partial [AR]} & \hspace{0.2cm} 2-2\dfrac{\partial \lambda_2 }{\partial [ARA]} \\ \dfrac{\partial \lambda_2 }{\partial [AR]} & \dfrac{\partial \lambda_2 }{\partial [ARA]} \end{bmatrix} \begin{bmatrix}\widetilde{k_{+}}A_{tot}\widetilde{R_{tot}}, \\ 0\end{bmatrix} \end{aligned}$$

4.27 $$\begin{aligned} \Longrightarrow \begin{bmatrix}k_{+}R_{tot}\\ 0\end{bmatrix}&= \widetilde{k_{+}} \widetilde{R_{tot}} \begin{bmatrix} \left( 1-2\dfrac{\partial \lambda_2 }{\partial [AR]} \right) \\ \dfrac{\partial \lambda_2 }{\partial [AR]} \end{bmatrix}. \end{aligned}$$

Equating the terms of the second row gives

4.28 $$\begin{aligned} \dfrac{\partial \lambda_2 }{\partial [AR]}=0. \end{aligned}$$

After substituting this into the equation given by the first row, we find that $k_{+}R_{tot}=\widetilde{k_{+}}\widetilde{R_{tot}}$, and so the first identifiable parameter combination is given by

4.29 $$\begin{aligned} C_{1} = k_{+}R_{tot}. \end{aligned}$$

The Jacobian matrix Eq. 4.25 can now be written as

4.30 $$\begin{aligned} \frac{\partial \boldsymbol{\lambda }}{\partial {\textbf {x}}}= \begin{bmatrix} 1 & \hspace{0.2cm}2-2\dfrac{\partial \lambda_2 }{\partial [ARA]} \\ 0 & \dfrac{\partial \lambda_2 }{\partial [ARA]} \end{bmatrix}. \end{aligned}$$

At this point, we see that if $\dfrac{\partial \lambda_2 }{\partial [ARA]} \not \equiv 0$, then the rank condition Eq. 2.16a is satisfied.

Next we consider condition Eq. 2.16c, which reads

4.31 $$\begin{aligned} {\textbf {f}}(\boldsymbol{\lambda }({\textbf {x}}),{\textbf {p}})=\dfrac{\partial \boldsymbol{\lambda }({\textbf {x}})}{\partial {\textbf {x}}} {\textbf {f}}({\textbf {x}},\widetilde{{\textbf {p}}}). \end{aligned}$$

Using the expression for $\lambda _{1}$ from Eq. 4.22, and the expression for $\textbf{f}$ from Eq. 4.13a, we find that the left-hand side of Eq. 4.31 is given by

4.32a $$\begin{aligned} {\textbf {f}}(\boldsymbol{\lambda }({\textbf {x}}),{\textbf {p}}) = \begin{bmatrix} b_{1}\lambda _{2}+c_{1} \\ b_{2} \lambda_{2}+c_{2} \end{bmatrix}, \end{aligned}$$

where

4.32b $$\begin{aligned} b_{1}&= k_{+}(1+2\alpha _+)(A_{tot}-[AR]-2[ARA]) + k_{-}(2+\alpha _-), \end{aligned}$$

4.32c $$\begin{aligned} c_{1}&= ([AR]+2[ARA])\Big (k_{+}(1+\alpha _+)([AR]+2[ARA]-A_{tot}) \\&- (k_{+}R_{tot}+k_{-})\Big ), \end{aligned}$$

4.32d $$\begin{aligned} b_{2}&= 2\alpha _+k_{+}\Big ([AR]+2[ARA]-A_{tot}\Big ) - \alpha _-k_{-}, \end{aligned}$$

4.32e $$\begin{aligned} c_{2}&= \alpha _+k_{+}\Big ([AR]+2[ARA]\Big )\Big (A_{tot}-[AR]-2[ARA]\Big ). \end{aligned}$$

The right-hand side of Eq. 4.31 is given by

4.33 $$\begin{aligned}&\dfrac{\partial \boldsymbol{\lambda }({\textbf {x}})}{\partial {\textbf {x}}} {\textbf {f}}({\textbf {x}},\widetilde{{\textbf {p}}}) = \nonumber \\&\begin{bmatrix} 1 & \quad 2-2\dfrac{\partial \lambda_{2} }{\partial [ARA]} \\ 0 & \dfrac{\partial \lambda_{2} }{\partial [ARA]} \end{bmatrix} \begin{bmatrix} \begin{aligned} & \Big \{\widetilde{k_{+}}(1 + \widetilde{\alpha _+})[AR]^2 + 2\widetilde{k_{+}}[ARA]^2 + \widetilde{k_{+}}(3 + 2\widetilde{\alpha _+})[AR][ARA]\Big . \\ & \qquad -\Big (\widetilde{k_{+}}(1+\widetilde{\alpha _+})A_{tot}+ \widetilde{k_{+}}\widetilde{R_{tot}} + \widetilde{k_{-}}\Big )[AR]\\ & \qquad \Big . + (\widetilde{\alpha _-}\widetilde{k_{-}} - \widetilde{k_{+}}A_{tot}- 2\widetilde{k_{+}}\widetilde{R_{tot}})[ARA]\Big \} \end{aligned} \\ -\widetilde{\alpha _+}\widetilde{k_{+}}[AR]^2 - 2\widetilde{\alpha _+}\widetilde{k_{+}}[AR][ARA]+ \widetilde{\alpha _+}\widetilde{k_{+}}A_{tot}[AR]- \widetilde{\alpha _-}\widetilde{k_{-}}[ARA]\end{bmatrix} . \end{aligned}$$

Equating the bottom row of Eq. 4.32a with the bottom row of Eq. 4.33 gives

4.34 $$\begin{aligned} \dfrac{\partial \lambda_{2} }{\partial [ARA]} = \frac{\begin{array}{c}\Bigg (2\alpha _+k_{+}\Big ([AR]+2[ARA]-A_{tot}\Big ) - \alpha _-k_{-}\Bigg ) \lambda_{2} \; + \\ \alpha _+k_{+}\Big ([AR]+2[ARA]\Big )\Big (A_{tot}-[AR]-2[ARA]\Big )\end{array}}{\begin{array}{c}-\widetilde{\alpha _+}\widetilde{k_{+}}[AR]^2 - 2\widetilde{\alpha _+}\widetilde{k_{+}}[AR][ARA]\\+ \widetilde{\alpha _+}\widetilde{k_{+}}A_{tot}[AR]- \widetilde{\alpha _-}\widetilde{k_{-}}[ARA]\end{array}}. \end{aligned}$$

Next, we equate the top row of Eq. 4.32a with the top row of Eq. 4.33 and substitute for $\dfrac{\partial \lambda_{2} }{\partial [ARA]}$ (given by Eq. 4.34) to give a linear equation for $\lambda _{2}$. Using MATLAB’s Symbolic Toolbox to aid with the lengthy algebraic manipulation, we find the following expression:

4.35 $$\begin{aligned} \lambda_{2} =\frac{m_{1}[AR]^2+ m_2[ARA]^{2} + m_{3}[AR][ARA]+ m_{4}[AR]+ m_{5}[ARA]}{k_{+}(2\alpha _+-1)\Big ([AR]+2[ARA]-A_{tot}\Big ) + k_{-}(2-\alpha _-)}, \end{aligned}$$

where

$$\begin{aligned} m_1&= \widetilde{k_{+}}(1-\widetilde{\alpha _+}) - k_{+}(1-\alpha _+), \\ m_2&= 2\widetilde{k_{+}} - 4k_{+}(1-\alpha _+), \\ m_3&= \widetilde{k_{+}}(3-2\widetilde{\alpha _+}) - 4k_{+}(1-\alpha _+), \\ m_4&= \widetilde{k_{+}}(\widetilde{\alpha _+}-1)A_{tot}-\widetilde{k_{-}} - k_{+}(\alpha _+-1)A_{tot}+ k_{-}, \\ m_5&= -\widetilde{\alpha _-}\widetilde{k_{-}} - \widetilde{k_{+}}A_{tot}- 2k_{+}(\alpha _+-1)A_{tot}+ 2k_{-}. \end{aligned}$$

Recall that we seek an analytic mapping (which is not unique) $\boldsymbol{\lambda }$. Given that $\dfrac{\partial \lambda_2 }{\partial [AR]}=0$, we therefore may write

$$\begin{aligned}\lambda_2 = \lambda_2 ([ARA]) = w_{0} + w_{1}[ARA] + w_{2}[ARA]^{2} + \cdots . \end{aligned}$$

Noting that the initial condition gives $\lambda _{2}(0)=0$, we see that

$$\begin{aligned} w_{0}=0, \end{aligned}$$

and hence the series expansion for $\lambda _{2}$ is given by

4.36 $$\begin{aligned}\lambda_2 = \lambda_2 ([ARA]) = w_{1}[ARA] + w_{2}[ARA]^{2} + \cdots . \end{aligned}$$

Equating the expansion in Eq. 4.36 with the expression for $\lambda_{2}$ in Eq. 4.35, we find that

4.37 $$\begin{aligned} m_{1} = 0, \qquad m_{4} = 0. \end{aligned}$$

Together, $m_{1}=0$ and $m_{4}=0$ give that

4.38 $$\begin{aligned} k_{-}= \widetilde{k_{-}}, \end{aligned}$$

and hence $k_{-}$ is identifiable.

Now we may rewrite Eq. 4.35 as

4.39 $$\begin{aligned} \lambda_{2} ([ARA]) =\frac{ \big (m_2[ARA]+ m_{3}[AR]+ m_{5}\big )\, [ARA]}{\begin{array}{c}2k_{+}(2\alpha _+-1)[ARA]\, + \, k_{+}(2\alpha _+-1)[AR]\,\\ + \, \big (k_{-}(2-\alpha _-)-k_{+}(2\alpha _+-1)A_{tot}\big )\end{array}}. \end{aligned}$$

The structure of the right-hand side of Eq. 4.39 suggests that a candidate mapping $\lambda _{2}$ may simply given by $\lambda _{2}([ARA])=[ARA]$. We now consider this candidate mapping and proceed to see whether it indeed results in an analytical mapping $\boldsymbol{\lambda }(\widetilde{{\textbf {x}}})$, from where we can apply the conditions in Eq. 2.16 to determine identifiability. For this choice of $\lambda _{2}$, Eq. 4.22 gives $\lambda _{1}([AR],[ARA])=[AR]$. Thus, we consider the mapping

4.40 $$\begin{aligned} \boldsymbol{\lambda }({\textbf {x}}) = {\textbf {x}}. \end{aligned}$$

The mapping $\boldsymbol{\lambda }$ is clearly analytic. For ease of calculation, we now revisit and apply the conditions Eq. 2.16, using the simple expression for $\boldsymbol{\lambda }$ in Eq. 4.40 together with the functions defined in Eq. 4.13.

We note that the Jacobian matrix is given by

4.41 $$\begin{aligned} \frac{\partial \boldsymbol{\lambda }}{\partial {\textbf {x}}}= \begin{bmatrix} 1 & 0 \\ 0 & 1 \end{bmatrix}. \end{aligned}$$

Condition Eq. 2.16a is satisfied by the rank-2 Jacobian, and condition Eq. 2.16b is satisfied since the initial condition is $$\textbf{x}_{0} = \begin{bmatrix} 0 \\ 0\end{bmatrix}$$.

Next, condition Eq. 2.16c gives that $f(\textbf{x},\textbf{p}) = f(\textbf{x},\widetilde{\textbf{p}})$. This leads to relationships between $\textbf{p}$ and $\widetilde{\textbf{p}}$ by considering coefficients of terms in Eq. 4.13a. We find that:

4.42a $$\begin{aligned} k_{+}(1+\alpha _+)&= \widetilde{k_{+}}(1+\widetilde{\alpha _+}), \end{aligned}$$

4.42b $$\begin{aligned} k_{+}&= \widetilde{k_{+}}, \end{aligned}$$

4.42c $$\begin{aligned} k_{+}(3+2\alpha _+)&= \widetilde{k_{+}}(3+2\widetilde{\alpha _+}), \end{aligned}$$

4.42d $$\begin{aligned} k_{+}(1+\alpha _+)A_{tot}+ k_{+}R_{tot}+ k_{-}&= \widetilde{k_{+}}(1+\widetilde{\alpha _+})A_{tot}+ \widetilde{k_{+}}\widetilde{R_{tot}}+ \widetilde{k_{-}}, \end{aligned}$$

4.42e $$\begin{aligned} \alpha _-k_{-}- k_{+}A_{tot}- 2k_{+}R_{tot}&= \widetilde{\alpha _-}\widetilde{k_{-}}- \widetilde{k_{+}}A_{tot}- 2\widetilde{k_{+}}\widetilde{R_{tot}}, \end{aligned}$$

4.42f $$\begin{aligned} \alpha _+k_{+}&= \widetilde{\alpha _+}\widetilde{k_{+}}, \end{aligned}$$

4.42g $$\begin{aligned} \alpha _-k_{-}&= \widetilde{\alpha _-}\widetilde{k_{-}}. \end{aligned}$$

Condition Eq. 2.16d gives that $g(\textbf{x},\textbf{p}) = g(\textbf{x},\widetilde{\textbf{p}})$. Considering Eq. 4.13b, we find that

4.42h $$\begin{aligned} k_{+}R_{tot}= \widetilde{k_{+}}\widetilde{R_{tot}}. \end{aligned}$$

Finally, condition Eq. 2.16e is satisfied by $\textbf{h}$ defined in Eq. 4.13c.

It is straightforward to show that the relations given in Eq. 4.42, together with Eq. 4.38, imply that

4.43a $$\begin{aligned} k_{+}&= \widetilde{k_{+}}, \end{aligned}$$

4.43b $$\begin{aligned} k_{-}&= \widetilde{k_{-}}, \end{aligned}$$

4.43c $$\begin{aligned} \alpha _+&= \widetilde{\alpha _+}, \end{aligned}$$

4.43d $$\begin{aligned} \alpha _-&= \widetilde{\alpha _-}, \end{aligned}$$

4.43e $$\begin{aligned} R_{tot}&= \widetilde{R_{tot}}. \end{aligned}$$

Substituting these relations in Eq. 4.43, together with $\lambda _{2}([ARA])=[ARA]$, into Eq. 4.34 gives $\dfrac{\partial \lambda_{2} }{\partial [ARA]} = 1$, as required by Eq. 4.40.

We conclude that all five parameters $k_{+}$, $k_{-}$, $\alpha _+$, $\alpha _-$ and $R_{tot}$ are identifiable.

## Ligand-induced dimerisation (LID)

Here we consider the ligand-induced dimerisation (LID) model analysed in [44]. LID is known to occur in the case of vascular endothelial growth factor (VEGF) receptors, and mis-regulated signalling via these receptors is implicated in angiogenesis and certain cancers [22, 24]. In the LID model [44], the binding of a bivalent ligand to one receptor monomer induces dimerisation with a neighbouring receptor monomer. The schematic for the model is given byIn this schematic, *R* represents a monomeric receptor, *AR* represents a ligand-bound monomeric receptor, and *RAR* represents ligand-bound dimerised receptor. Here, $k_{+}$ and $k_{-}$ are the kinetic association and dissociation rate constants. Also, $\psi =\psi _{+}/\psi _{-}$ is the equilibrium cooperativity factor, which measures the ligand’s changed affinity for the second receptor monomer as a result of being bound to the first monomer.

### Model formulation

The ODE system that governs the system dynamics is given by

5.1a $$\begin{aligned} \frac{d[R]}{dt}=&- k_{+}[A][R]+ k_{-}[AR]- \psi _+k_{+}[R][AR]+ \psi _-k_{-}[RAR], \end{aligned}$$

5.1b $$\begin{aligned} \frac{d[AR]}{dt}=&\ k_{+}[A][R]- k_{-}[AR]- \psi _+k_{+}[R][AR]+ \psi _-k_{-}[RAR], \end{aligned}$$

5.1c $$\begin{aligned} \frac{d[RAR]}{dt}=&\ \psi _+k_{+}[R][AR]- \psi _-k_{-}[RAR], \end{aligned}$$

with initial conditions

5.1d $$\begin{aligned} [R](0)=R_{tot},\qquad [AR](0)=0,\qquad [RAR](0)=0. \end{aligned}$$

The signal of interest, and measured output for the system, is proportional to the concentration of bound receptors, that is

5.1e $$\begin{aligned} y=a([AR]+2[RAR]), \end{aligned}$$

for an experimental scaling factor *a*. This approach allows us to both (i) align with the model given in [44], and (ii) provide an example which contrasts with the analyses in Sections“Ligand binding monomeric receptor with ligand depletion” and“Ligand binding pre-dimerised receptor with ligand depletion”, where the *concentrations* were considered as readouts.

In [44], parameter estimates are obtained for three different VEGF receptor ligands by fitting to experimental timecourses, with excellent agreement between model and data. The estimates are noted to be plausible by comparison with previously published values, and the good fits across all data sets represent a preliminary validation of the model, but the question of identifiability remains. We now address this by applying SIA to the model.

Assuming that there is no ligand depletion, as we did in [44], the ligand concentration [*A*] is constant, and this is the only known parameter in the model. Therefore, the vector containing all unknown parameters is given by ${\textbf {p}}=(a,k_{+},k_{-},\psi _+,\psi _-,R_{tot})$. We note that the system Eq. 5.1 is nonlinear, which reduces the number of available methods to determine identifiability. The transfer function method used for other receptor binding scenarios in [45] is applicable only to linear systems, and thus cannot be used this time.

### SIA for LID model - Taylor series

We first consider application of the Taylor series method that we introduced in Section“Taylor series method” to the LID model Eq. 5.1. The calculations are shown in Appendix E. Similarly to the Taylor series method for pre-dimerised receptors with ligand depletion, the Taylor coefficients become unwieldy and there is no clear systematic approach for using the coefficients to determine identifiability. Any progress would rely on an ad hoc approach and lengthy symbolic computation. These issues were noted in Section“Ligand binding pre-dimerised receptor with ligand depletion” and also [4]. Again, we choose not to pursue the Taylor series method for the LID model.

### SIA for LID model - similarity transformation

As in Section“SIA for ligand depletion dimer model - similarity transformation”, we first use a conservation law to reduce the dimension of the governing system (to aid the controllability and observability checks). Total receptor is conserved, such that

5.2 $$\begin{aligned} [R]+[AR]+2[RAR]= R_{tot}, \end{aligned}$$

where $R_{tot}$ is the total receptor concentration (initially all in monomeric form). Then we may eliminate [*R*] and rewrite Eq. 5.1 in reduced form as follows:

5.3a $$\begin{aligned} \frac{d[AR]}{dt}&= \psi _+k_{+}[AR]^2 + 2\psi _+k_{+}[AR][RAR]- (k_{+}[A]+k_{-}+ \psi _+k_{+}R_{tot})[AR] \nonumber \\&\qquad + (\psi _-k_{-}- 2k_{+}[A])[RAR] + k_{+}[A]R_{tot}, \end{aligned}$$

5.3b $$\begin{aligned} \frac{d[RAR]}{dt}&= -\psi _+k_{+}[AR]^2 - 2\psi _+k_{+}[AR][RAR]\\&+ \psi _+k_{+}R_{tot}[AR] - \psi _-k_{-}[RAR], \end{aligned}$$

with initial conditions

5.3c $$\begin{aligned} [AR](0)=0,\qquad [RAR](0)=0, \end{aligned}$$

and again the output

5.3d $$\begin{aligned} y=a([AR]+2[RAR]). \end{aligned}$$

In the form of system Eq. 2.1, this model corresponds to

5.4a $$\begin{aligned} \textbf{f}&= \begin{bmatrix} \begin{aligned} & \psi _+k_{+}[AR]^2 + 2\psi _+k_{+}[AR][RAR]- (k_{+}[A]+k_{-}+ \psi _+k_{+}R_{tot})[AR] \\ & \; + (\psi _-k_{-}- 2k_{+}[A])[RAR] \end{aligned} \\ -\psi _+k_{+}[AR]^2 - 2\psi _+k_{+}[AR][RAR]+ \psi _+k_{+}R_{tot}[AR] - \psi _-k_{-}[RAR] \end{bmatrix}, \end{aligned}$$

5.4b $$\begin{aligned} \textbf{g}&= \begin{bmatrix} k_{+}[A]R_{tot}\\ 0 \end{bmatrix}, \end{aligned}$$

5.4c $$\begin{aligned} y&= a([AR]+2[RAR]), \end{aligned}$$

5.4d $$\begin{aligned} h&= a[1 \;\; 2]\, \textbf{x}, \end{aligned}$$

5.4e $$\begin{aligned} \textbf{x}&= \begin{bmatrix} [AR] \\ [RAR] \end{bmatrix},\end{aligned}$$

5.4f $$\begin{aligned} \textbf{x}_{0}&= \begin{bmatrix} 0 \\ 0 \end{bmatrix}, \end{aligned}$$

where $$n_{x}=2$$.

#### Observability and controllability rank conditions

First, we check the observability rank condition by making use of the Lie derivative and observability matrix that were defined in Eqs. 2.10-2.12. For $n_{x}=2$, we construct the observability matrix $\mathcal {O}$, given by

5.5 $$\begin{aligned} \mathcal {O}=\begin{bmatrix} \dfrac{\partial L^0_{\textbf {f}}y({\textbf {x)}}}{\partial {\textbf {x}}} \\ \dfrac{\partial L^1_{\textbf {f}}y({\textbf {x)}}}{\partial {\textbf {x}}} \end{bmatrix}. \end{aligned}$$

Recall that

5.6 $$\begin{aligned} L_{\textbf {f}}^0 y({\textbf {x}})=y({\textbf {x}}), \qquad L^1_{\textbf {f}}y({\textbf {x}})=\frac{\partial y({\textbf {x}})}{\partial {\textbf {x}}} {\textbf {f}}({\textbf {x}}). \end{aligned}$$

For our model, we have

5.7 $$\begin{aligned} L_{\textbf {f}}^0 y({\textbf {x}}) = a[1 \;\; 2]\textbf{x}, \;\; \Longrightarrow \;\; \dfrac{\partial L^0_{\textbf {f}}y({\textbf {x)}}}{\partial {\textbf {x}}} = a[1 \;\; 2]. \end{aligned}$$

Also, we have

5.8 $$\begin{aligned} L_{\textbf {f}}^1 y({\textbf {x}})&= \frac{\partial y({\textbf {x}})}{\partial {\textbf {x}}} {\textbf {f}}({\textbf {x}}) \nonumber \\&= a\Big \{ -\psi _+k_{+}[AR]^2 - 2\psi _+k_{+}[AR][ARA]-(k_{+}[A]+k_{-}\nonumber \\&\qquad \qquad- \psi _+k_{+}R_{tot})[AR]\Big . \Big . -(\psi _-k_{-}+2k_{+}[A])[RAR\Big \}, \end{aligned}$$

which gives

5.9 $$\begin{aligned}&\small \dfrac{\partial L^1_{\textbf {f}}y({\textbf {x)}}}{\partial {\textbf {x}}} \\&= a \begin{bmatrix} \begin{array}{c} -\psi _+k_{+}\big (2([AR]+[RAR]) - R_{tot}\big ) \\- (k_{+}[A]+k_{-})\end{array} & -\big (\begin{array}{c}2\psi _+k_{+}[AR]+\psi _-k_{-}\\+2k_{+}[A]\end{array}\big ) \end{bmatrix}\end{aligned} .$$

This results in the following observability matrix:

5.10 $$ \mathcal {O}=a \begin{bmatrix} 1 & 2 \\ -\begin{array}{c}\psi _+k_{+}\big (2([AR]+[RAR])\\ - R_{tot}\big ) - (k_{+}[A]+k_{-})\end{array} & -\big (2\psi _+k_{+}[AR]+\psi _-k_{-}+2k_{+}[A]\big ) \end{bmatrix} .$$

We note that $\textrm{rank}(\mathcal {O})=n_{x}=2$, so the observability rank criterion is satisfied.

Next, we determine the controllability matrix, which was defined in Eq. 2.9, using the Lie bracket. For $n_{x}=2$, we construct the controllability matrix $\mathcal {C}$, given by

5.11 $$\begin{aligned} \mathcal {C}=[{\textbf {g}},(ad^1_{\textbf {f}},{\textbf {g}})], \end{aligned}$$

where we recall that (see Eq. 2.7)

$$\begin{aligned} (ad^1_{\textbf {f}},{\textbf {g}})=[{\textbf {f}},{\textbf {g}}]=\frac{\partial {\textbf {g}}({\textbf {x}})}{\partial {\textbf {x}}} {\textbf {f}}({\textbf {x}})-\frac{\partial {\textbf {f}}({\textbf {x}})}{\partial {\textbf {x}}} {\textbf {g}}({\textbf {x}}). \end{aligned}$$

For our model, $\textbf{f}$ is given by Eq. 5.4a and $\textbf{g}$ is given by Eq. 5.4b, so that

$$\begin{aligned} \frac{\partial {\textbf {g}}({\textbf {x}})}{\partial {\textbf {x}}} = \begin{bmatrix} 0 & 0 \\ 0 & 0 \end{bmatrix}, \end{aligned}$$

and

$$\begin{aligned} \frac{\partial {\textbf {f}}({\textbf {x}})}{\partial {\textbf {x}}} = \begin{bmatrix} \hat{J}_{11} & \hat{J}_{12} \\ \hat{J}_{21} & \hat{J}_{22} \end{bmatrix}, \end{aligned}$$

where

$$\begin{aligned} \hat{J}_{11}&= \psi _+k_{+}\big (2[AR]+2[RAR]- R_{tot}) - (k_{+}[A]+k_{-}) , \\ \hat{J}_{12}&= 2\psi _+k_{+}[AR]+ \psi _-k_{-}- 2k_{+}[A], \\ \hat{J}_{21}&= -\psi _+k_{+}\big (2[AR]+2[RAR]- R_{tot}), \\ \hat{J}_{22}&= -2\psi _+k_{+}[AR]- \psi _-k_{-}. \end{aligned}$$

Therefore the controllability matrix $\mathcal {C}$ is given by

5.12 $$\begin{aligned} \mathcal {C}= k_{+}[A]R_{tot}\begin{bmatrix} 1 & -\hat{J}_{11} \\ 0 & -\hat{J}_{21} \end{bmatrix}, \end{aligned}$$

which has $\textrm{rank}(\mathcal {C})=n_{x} = 2$, so the controllability rank criterion is satisfied. Thus the system is both observable and controllable, and we proceed with the similarity transformation method.

#### Applying the method

All conditions in Eq. 2.16 should be satisfied, and we can consider them simultaneously. Here, we subsequently assume them to hold in an order that gives the most immediate results. We assume that there exists an analytical mapping $\boldsymbol{\lambda }({\textbf {x}})=[\lambda _1({\textbf {x}}),\lambda_{2}({\textbf {x}})]^{T}$, then we first assume that condition Eq. 2.16e holds, such that $h(\boldsymbol{\lambda }({\textbf {x}}),{\textbf {p}})=h({\textbf {x}},\widetilde{{\textbf {p}}})$. This gives

5.13 $$\begin{aligned} a(\lambda _1+2\lambda_{2} )=\widetilde{a}([AR]+2[RAR]),\qquad \Rightarrow \qquad \lambda _1=\frac{\widetilde{a}}{a}([AR]+2[RAR])-2\lambda_{2} . \end{aligned}$$

We next construct the Jacobian

$$\begin{aligned} \displaystyle {\frac{\partial \boldsymbol{\lambda }}{\partial {\textbf {x}}} = \begin{bmatrix} \frac{\partial \lambda _1}{\partial [AR]} & \frac{\partial \lambda _1}{\partial [ARA]} \\ \frac{\partial \lambda_2 }{\partial [AR]} & \frac{\partial \lambda_2 }{\partial [ARA]} \end{bmatrix}.} \end{aligned}$$

Differentiating Eq. 5.13 with respect to *[AR]* gives

5.14 $$\begin{aligned} \frac{\partial \lambda _1}{\partial [AR]}=\frac{\widetilde{a}}{a}-2\frac{\partial \lambda_2 }{\partial [AR]}. \end{aligned}$$

Similarly, differentiating Eq. 5.13 with respect to *[RAR]* gives

5.15 $$\begin{aligned} \frac{\partial \lambda _1}{\partial [RAR]}=2\left( \frac{\widetilde{a}}{a}-\frac{\partial \lambda_2 }{\partial [RAR]}\right) . \end{aligned}$$

Hence the Jacobian of $\boldsymbol{\lambda }({\textbf {x}})$ is given by

5.16 $$\begin{aligned} \frac{\partial \boldsymbol{\lambda }}{\partial {\textbf {x}}}=\begin{bmatrix} \dfrac{\widetilde{a}}{a}-2\dfrac{\partial \lambda_2 }{\partial [AR]} & 2\left( \dfrac{\widetilde{a}}{a}-\dfrac{\partial \lambda_2 }{\partial [RAR]}\right) \\ \dfrac{\partial \lambda_2 }{\partial [AR]} & \dfrac{\partial \lambda_2 }{\partial [RAR]} \end{bmatrix}. \end{aligned}$$

We next consider condition Eq. 2.16d, namely

$$\begin{aligned} {\textbf {g}}(\boldsymbol{\lambda }({\textbf {x}}),{\textbf {p}})=\dfrac{\partial \boldsymbol{\lambda }({\textbf {x}})}{\partial {\textbf {x}}} {\textbf {g}}({\textbf {x}},\widetilde{{\textbf {p}}}), \end{aligned}$$

and use expression Eq. 5.16 to find that

5.17 $$\begin{aligned} \begin{bmatrix}k_{+}[A]R_{tot}\\ 0 \end{bmatrix}&= \begin{bmatrix} \dfrac{\widetilde{a}}{a}-2\dfrac{\partial \lambda_2 }{\partial [AR]} & \hspace{.5ex}2\left( \dfrac{\widetilde{a}}{a}-\dfrac{\partial \lambda_2 }{\partial [RAR]}\right) \\ \dfrac{\partial \lambda_2 }{\partial [AR]} & \dfrac{\partial \lambda_2 }{\partial [RAR]} \end{bmatrix} \begin{bmatrix}\widetilde{k_{+}}[A]\widetilde{R_{tot}} \\ 0\end{bmatrix}, \end{aligned}$$

5.18 $$\begin{aligned} \Longrightarrow \;\; \begin{bmatrix}k_{+}R_{tot}\\ 0\end{bmatrix}&= \widetilde{k_{+}}\widetilde{R_{tot}} \begin{bmatrix} \left( \dfrac{\widetilde{a}}{a}-2\dfrac{\partial \lambda_2 }{\partial [AR]} \right) \\ \dfrac{\partial \lambda_2 }{\partial [AR]} \end{bmatrix}. \end{aligned}$$

Equating the terms of the second row gives

5.19 $$\begin{aligned} \dfrac{\partial \lambda_2 }{\partial [AR]}=0. \end{aligned}$$

After substituting this into the equation given by the first row, we find that $ak_{+}R_{tot}=\widetilde{a}\widetilde{k_{+}}\widetilde{R_{tot}}$, and so the first identifiable parameter combination $C_{1}$ is given by

5.20 $$\begin{aligned} C_{1}=ak_{+}R_{tot}, \end{aligned}$$

which is the same conclusion that we drew from Eq. E.4. The Jacobian matrix Eq. 5.16 can now be written as

5.21 $$\begin{aligned} \frac{\partial \boldsymbol{\lambda }}{\partial {\textbf {x}}}=\begin{bmatrix} \dfrac{\widetilde{a}}{a} & \hspace{0.5ex}2\left( \dfrac{\widetilde{a}}{a}-\dfrac{\partial \lambda_2 }{\partial [RAR]}\right) \\ 0 & \dfrac{\partial \lambda_2 }{\partial [RAR]} \end{bmatrix}. \end{aligned}$$

At this point, we see that we need that $\dfrac{\partial \lambda_2 }{\partial [ARA]} \not \equiv 0$ in order for the rank condition Eq. 2.16a to be satisfied.

Next we consider condition Eq. 2.16c, which reads

5.22 $$\begin{aligned} {\textbf {f}}(\boldsymbol{\lambda }({\textbf {x}}),{\textbf {p}})=\dfrac{\partial \boldsymbol{\lambda }({\textbf {x}})}{\partial {\textbf {x}}} {\textbf {f}}({\textbf {x}},\widetilde{{\textbf {p}}}). \end{aligned}$$

Using the expression for $\lambda _{1}$ from Eq. 5.13, and the expression for $\textbf{f}$ from Eq. 5.4a, we find that the left-hand side of Eq. 5.22 is given by

5.23a $$\begin{aligned} {\textbf {f}}(\boldsymbol{\lambda }({\textbf {x}}),{\textbf {p}}) = \begin{bmatrix} \beta _{1}\lambda _{2}+\gamma _{1} \\ \beta _{2}\lambda _{2}+\gamma _{2} \end{bmatrix}, \end{aligned}$$

where

5.23b $$\begin{aligned} \beta _{1}&= -2\psi _{+}k_{+}\left( \frac{\widetilde{a}}{a}([AR]+2[RAR]) - R_{tot}\right) + k_{-}(2+\psi _{-}), \end{aligned}$$

5.23c $$\begin{aligned} \gamma _{1}&= \frac{\widetilde{a}}{a}([AR]+2[RAR])\Big (\psi _{+}k_{+}\frac{\widetilde{a}}{a}([AR]+2[RAR])\nonumber \\ & \quad-k_{+}[A]-k_{-}-\psi _{+}k_{+}R_{tot}\Big ), \end{aligned}$$

5.23d $$\begin{aligned} \beta _{2}&= 2\psi _{+}k_{+}\Big (\frac{\widetilde{a}}{a}([AR]+2[RAR]) - R_{tot}\Big ) - \psi _{-}k_{-}, \end{aligned}$$

5.23e $$\begin{aligned} \gamma _{2}&= \frac{\widetilde{a}}{a}\psi _{+}k_{+}([AR]+2[RAR]) \Big (R_{tot}- \frac{\widetilde{a}}{a}([AR]+2[RAR])\Big ). \end{aligned}$$

The right-hand side of Eq. 5.22 is given by

5.24 $$\begin{aligned}&\dfrac{\partial \boldsymbol{\lambda }({\textbf {x}})}{\partial {\textbf {x}}} {\textbf {f}}({\textbf {x}},\widetilde{{\textbf {p}}}) = \nonumber \\&\begin{bmatrix} \dfrac{\widetilde{a}}{a} & 2\left( \dfrac{\widetilde{a}}{a}-\dfrac{\partial \lambda_2 }{\partial [RAR]}\right) \\ 0 & \dfrac{\partial \lambda_2 }{\partial [RAR]} \end{bmatrix} \begin{bmatrix}\begin{aligned} \widetilde{\psi _+}& \widetilde{k_{+}}([AR]+2[RAR])[AR]\\ & -(\widetilde{k_{+}}[A]+\widetilde{k_{-}}+\widetilde{\psi _+}\widetilde{k_{+}}\widetilde{R_{tot}})[AR]+(\widetilde{\psi _-}\widetilde{k_{-}}-2\widetilde{k_{+}}[A])[RAR]\end{aligned} \\ -\widetilde{\psi _+}\widetilde{k_{+}}[AR]^2-2\widetilde{\psi _+}\widetilde{k_{+}}[AR][RAR]+\widetilde{\psi _+}\widetilde{k_{+}}\widetilde{R_{tot}}[AR]-\widetilde{\psi _-}\widetilde{k_{-}}[RAR]\end{bmatrix}. \end{aligned}$$

Equating the bottom row of Eq. 5.23a with the bottom row of Eq. 5.24, and rewriting, gives

5.25 $$\begin{aligned}&\dfrac{\partial \lambda_2 }{\partial [RAR]} = \nonumber \\&\frac{\begin{array}{c}\Bigg (2\psi _{+}k_{+}\Big (\frac{\widetilde{a}}{a}([AR]+2[RAR]) - R_{tot}\Big ) - \psi _{-}k_{-}\Bigg )\\ \lambda_{2} \; + \; \frac{\widetilde{a}}{a}\psi _{+}k_{+}([AR]+2[RAR]) \Big (R_{tot}- \frac{\widetilde{a}}{a}([AR]+2[RAR])\Big )\end{array}}{\begin{array}{c}-\widetilde{\psi _+}\widetilde{k_{+}}[AR]^2-2\widetilde{\psi _+}\widetilde{k_{+}}[AR][RAR]\\+\widetilde{\psi _+}\widetilde{k_{+}}\widetilde{R_{tot}}[AR]-\widetilde{\psi _-}\widetilde{k_{-}}[RAR]\end{array}}. \end{aligned}$$

Next, we equate the top row of Eq. 5.23a with the top row of Eq. 5.24 and substitute the expression Eq. 5.25 that we determined for $\dfrac{\partial \lambda_2 }{\partial [RAR]}$ to give a linear equation for $\lambda _{2}$. Using MATLAB’s Symbolic Toolbox to aid with the lengthy algebraic manipulation, we find the following expression:

5.26 $$\begin{aligned} \lambda_{2} =\frac{M_{1}[AR]^2+ M_2[RAR]^{2} + M_{3}[AR][RAR]+ M_{4}[AR]+ M_{5}[RAR]}{-2\frac{\widetilde{a}}{a}\psi _{+}k_{+}[AR]- 4 \frac{\widetilde{a}}{a}\psi _{+}k_{+}[RAR]+ (\psi _{-}k_{-}- 2k_{-}+ 2\psi _{+}k_{+}R_{tot})}, \end{aligned}$$

where

$$\begin{aligned} M_1&= \frac{\widetilde{a}}{a}\left( \widetilde{\psi _{+}}\widetilde{k_{+}}-\frac{\widetilde{a}}{a}\psi _{+}k_{+}\right) , \\ M_2&= -4\left( \frac{\widetilde{a}}{a}\right) ^{2} \psi _{+}k_{+}, \\ M_3&= 2\frac{\widetilde{a}}{a}\left( \widetilde{\psi _{+}}\widetilde{k_{+}}- 2\frac{\widetilde{a}}{a}\psi _{+}k_{+}\right) , \\ M_4&= -\frac{\widetilde{a}}{a}\Big (k_{-}-\widetilde{k_{-}}+ k_{+}[A]- \widetilde{k_{+}}[A]+ \widetilde{\psi _{+}}\widetilde{k_{+}}\widetilde{R_{tot}}- \psi _{+}k_{+}R_{tot}\Big ), \\ M_5&= \frac{\widetilde{a}}{a}\Big (2\widetilde{k_{+}}[A]-2k_{+}[A]-2k_{-}+ \widetilde{\psi _{-}}\widetilde{k_{-}}+ 2\psi _{+}k_{+}R_{tot}\Big ). \end{aligned}$$

Recall that we seek an analytic mapping $\boldsymbol{\lambda }$ (which is not necessarily unique). Given that $\dfrac{\partial \lambda_2 }{\partial [AR]}=0$, we therefore may write

$$\begin{aligned}\lambda_2 = \lambda_2 ([RAR]) = \eta _{0} + \eta _{1}[RAR] + \eta _{2}[RAR]^{2} + \cdots . \end{aligned}$$

Applying condition Eq. 2.16b together with the initial condition Eq. 5.4f gives

$$\begin{aligned} \eta _{0}=0, \end{aligned}$$

and hence the series expansion for $\lambda _{2}$ is given by

5.27 $$\begin{aligned} \lambda_2 = \lambda_2 ([RAR]) = \eta _{1}[RAR] + \eta _{2}[RAR]^{2} + \cdots . \end{aligned}$$

Equating the expansion in Eq. 5.27 with the expression for $\lambda_{2}$ in Eq. 5.26, we find that

5.28 $$\begin{aligned} M_{1} = 0, \qquad M_{4} = 0. \end{aligned}$$

We note that $M_{1}=0$ gives

5.29 $$\begin{aligned} \frac{\widetilde{\psi _{+}}\widetilde{k_{+}}}{\widetilde{a}} = \frac{\psi _{+}k_{+}}{a}, \end{aligned}$$

and hence

5.30 $$\begin{aligned} C_{2} = \frac{\psi _{+}k_{+}}{a} \end{aligned}$$

is identifiable. Also, $M_{4}=0$ gives

5.31 $$\begin{aligned} \widetilde{k_{-}}+\widetilde{k_{+}}[A]-\widetilde{\psi _{+}}\widetilde{k_{+}}\widetilde{R_{tot}}= k_{-}+k_{+}[A]-\psi _{+}k_{+}R_{tot}, \end{aligned}$$

and hence

5.32 $$\begin{aligned} C_{3} = k_{-}+k_{+}[A]-\psi _{+}k_{+}R_{tot}, \end{aligned}$$

is identifiable.

Taking into account the relationship Eq. 5.29, we see that $M_{3}$ may now be written

5.33 $$\begin{aligned} M_{3} = -2\left( \frac{\widetilde{a}}{a}\right) ^{2} \psi _{+}k_{+}. \end{aligned}$$

Also, using the fact that $ak_{+}R_{tot}=\widetilde{a}\widetilde{k_{+}}\widetilde{R_{tot}}$ (which led to Eqs. 5.20) and 5.31, we find that $M_{5}$ can now be written as

5.34 $$\begin{aligned} M_{5} = \frac{\widetilde{a}}{a}(\widetilde{\psi _{-}}\widetilde{k_{-}}- 2\widetilde{k_{-}}+ 2\widetilde{\psi _{+}}\widetilde{k_{+}}\widetilde{R_{tot}}). \end{aligned}$$

Next we rewrite Eq. 5.26 as

5.35 $$\begin{aligned}&\lambda_{2} = \lambda_{2} ([RAR]) \\&= \frac{\begin{array}{c}\frac{\widetilde{a}}{a}[RAR]\Big (-4\frac{\widetilde{a}}{a}\psi _{+}k_{+}[RAR]- 2\frac{\widetilde{a}}{a}\psi _{+}k_{+}[AR]\\+ (\widetilde{\psi _{-}}\widetilde{k_{-}}- 2\widetilde{k_{-}}+ 2\widetilde{\psi _{+}}\widetilde{k_{+}}\widetilde{R_{tot}})\Big )\end{array}}{\begin{array}{c}-4\frac{\widetilde{a}}{a}\psi _{+}k_{+}[RAR]- 2\frac{\widetilde{a}}{a}\psi _{+}k_{+}[AR]\\+ (\psi _{-}k_{-}- 2k_{-}+ 2\psi _{+}k_{+}R_{tot})\end{array}}. \end{aligned}$$

Equation 5.35 suggests a candidate mapping

5.36 $$\begin{aligned} \lambda _{2}([RAR])=\frac{\widetilde{a}}{a}[RAR], \end{aligned}$$

due to the similarity between the denominator and the factor in large brackets in the numerator. We now consider this candidate mapping and proceed to see whether it indeed results in an analytical mapping $\boldsymbol{\lambda }(\widetilde{{\textbf {x}}})$, from where we can apply the conditions in Eq. 2.16 to determine identifiability. First note that this mapping requires that

$$\begin{aligned} \psi _{-}k_{-}- 2k_{-}+ 2\psi _{+}k_{+}R_{tot}= \widetilde{\psi _{-}}\widetilde{k_{-}}- 2\widetilde{k_{-}}+ 2\widetilde{\psi _{+}}\widetilde{k_{+}}\widetilde{R_{tot}}, \end{aligned}$$

so that

5.37 $$\begin{aligned} C_{4} = \psi _{-}k_{-}- 2k_{-}+ 2\psi _{+}k_{+}R_{tot}\end{aligned}$$

is identifiable.

Together, Eqs. 5.13 and 5.36 give

5.38 $$\begin{aligned} \lambda _{1}([AR],[RAR]) = \frac{\widetilde{a}}{a}[AR], \end{aligned}$$

and hence

5.39 $$\begin{aligned} \boldsymbol{\lambda }({\textbf {x}}) = \frac{\widetilde{a}}{a}{\textbf {x}}. \end{aligned}$$

Then the Jacobian becomes

5.40 $$\begin{aligned} \displaystyle {\frac{\partial \boldsymbol{\lambda }}{\partial {\textbf {x}}} = \frac{\widetilde{a}}{a}\begin{bmatrix} 1 & 0 \\ 0 & 1 \end{bmatrix}.} \end{aligned}$$

It is clear that condition Eq. 2.16a is satisfied by the rank-2 Jacobian.

We may now use Eq. 5.39 to simplify the parameter relationships arising from condition Eq. 2.16c. We see that Eq. 2.16c gives that

5.41 $$\begin{aligned} f(\frac{\widetilde{a}}{a}\textbf{x},\textbf{p}) = \frac{\widetilde{a}}{a}f(\textbf{x},\widetilde{\textbf{p}}). \end{aligned}$$

This leads to relationships between $\textbf{p}$ and $\widetilde{\textbf{p}}$ by considering coefficients of terms in Eq. 5.4a. Comparing the coefficients of $[AR]^{2}$, [*AR*][*RAR*], [*AR*] and [*RAR*] in the top row gives:

5.42a $$\begin{aligned} \frac{\widetilde{a}}{a}\psi _{+}k_{+}&= \widetilde{\psi _{+}}\widetilde{k_{+}}, \end{aligned}$$

5.42b $$\begin{aligned} \frac{\widetilde{a}}{a}\psi _{+}k_{+}&= \widetilde{\psi _{+}}\widetilde{k_{+}}, \end{aligned}$$

5.42c $$\begin{aligned} k_{+}[A]+ k_{-}+ \psi _{+}k_{+}R_{tot}&= \widetilde{k_{+}}[A]+ \widetilde{k_{-}}+ \widetilde{\psi _{+}}\widetilde{k_{+}}\widetilde{R_{tot}}, \end{aligned}$$

5.42d $$\begin{aligned} \psi _{-}k_{-}- 2k_{+}[A]&= \widetilde{\psi _{-}}\widetilde{k_{-}}- 2\widetilde{k_{+}}[A]. \end{aligned}$$

The first two of these equations give no new information, since we already found Eqs. 5.30. Using Eqs. 5.42c and 5.32 together gives

5.43 $$\begin{aligned} \psi _{+}k_{+}R_{tot}= \widetilde{\psi _{+}}\widetilde{k_{+}}\widetilde{R_{tot}}, \end{aligned}$$

revealing another identifiable combination

5.44 $$\begin{aligned} C_{5} = \psi _{+}k_{+}R_{tot}. \end{aligned}$$

Using the expressions for $C_{1}$ and $C_{2}$ from Eqs. 5.20 and 5.30, we see that

$$\begin{aligned} k_{+} = \frac{C_{1}C_{2}}{C_{5}}, \;\; \Rightarrow \;\; k_{+}= \widetilde{k_{+}}, \end{aligned}$$

and hence $k_{+}$ is identifiable. Then from Eq. 5.42d, we see that

$$\begin{aligned} \psi _{-}k_{-}=\widetilde{\psi _{-}}\widetilde{k_{-}}, \end{aligned}$$

and hence

5.45 $$\begin{aligned} C_{6} = \psi _{-}k_{-}\end{aligned}$$

is identifiable. Considering Eqs. 5.37, 5.44 and 5.45 together, we see that

5.46 $$\begin{aligned} k_{-} = \frac{1}{2}\left( C_{6}-C_{4}+2C_{5}\right) , \;\; \Rightarrow \;\; k_{-}= \widetilde{k_{-}}, \end{aligned}$$

and hence $k_{-}$ is identifiable. Then from Eq. 5.45 we find that $\psi _{-}$ is also identifiable.

By comparing the coefficients of $[AR]^{2}$, [*AR*][*RAR*], [*AR*] and [*RAR*] in the top row of Eq. 5.41 (using Eq. 5.4a), we have found that $k_{+}$, $k_{-}$ and $\psi _{-}$ are all identifiable, in addition to the identifiable combinations $C_{1}$ and $C_{2}$ given by Eq. 5.20 and Eq. 5.30. In summary, the following five parameters and combinations are identifiable:

5.47a $$\begin{aligned} k_{+}&= \widetilde{k_{+}}, \end{aligned}$$

5.47b $$\begin{aligned} k_{-}&= \widetilde{k_{-}}, \end{aligned}$$

5.47c $$\begin{aligned} \psi _{-}&= \widetilde{\psi _{-}}, \end{aligned}$$

5.47d $$\begin{aligned} aR_{tot}&= \widetilde{a}\widetilde{R_{tot}}, \end{aligned}$$

5.47e $$\begin{aligned} \frac{\psi _{+}}{a}&= \frac{\widetilde{\psi _{+}}}{\widetilde{a}}. \end{aligned}$$

Comparing the coefficients of $[AR]^{2}$, [*AR*][*RAR*], [*AR*] and [*RAR*] in the bottom row of Eq. 5.4a gives no further information.

We note that condition Eq. 2.16e is satisfied by $\textbf{h}$ defined in Eq. 5.4c and the mapping Eq. 5.39, and reveals no further identifiable combinations.

We have found an analytical mapping $\boldsymbol{\lambda }({\textbf {x}})$ given by Eq. 5.39 for which the relationships in Eq. 5.47 follow from the conditions in Eq. 2.16. We conclude that the similarity transformation approach has revealed that the five parameters and combinations listed in Eq. 5.47 are identifiable. In particular, from the original system parameters, $k_{+}$, $k_{-}$ and $\psi _{-}$ are uniquely identifiable, while $R_{tot}$, $\psi _{+}$ and *a* are not. It is worth noting that the on and off rates are uniquely identifiable, and these are often the primary kinetic parameters of interest.

### Numerical results

**Fig. 3:**
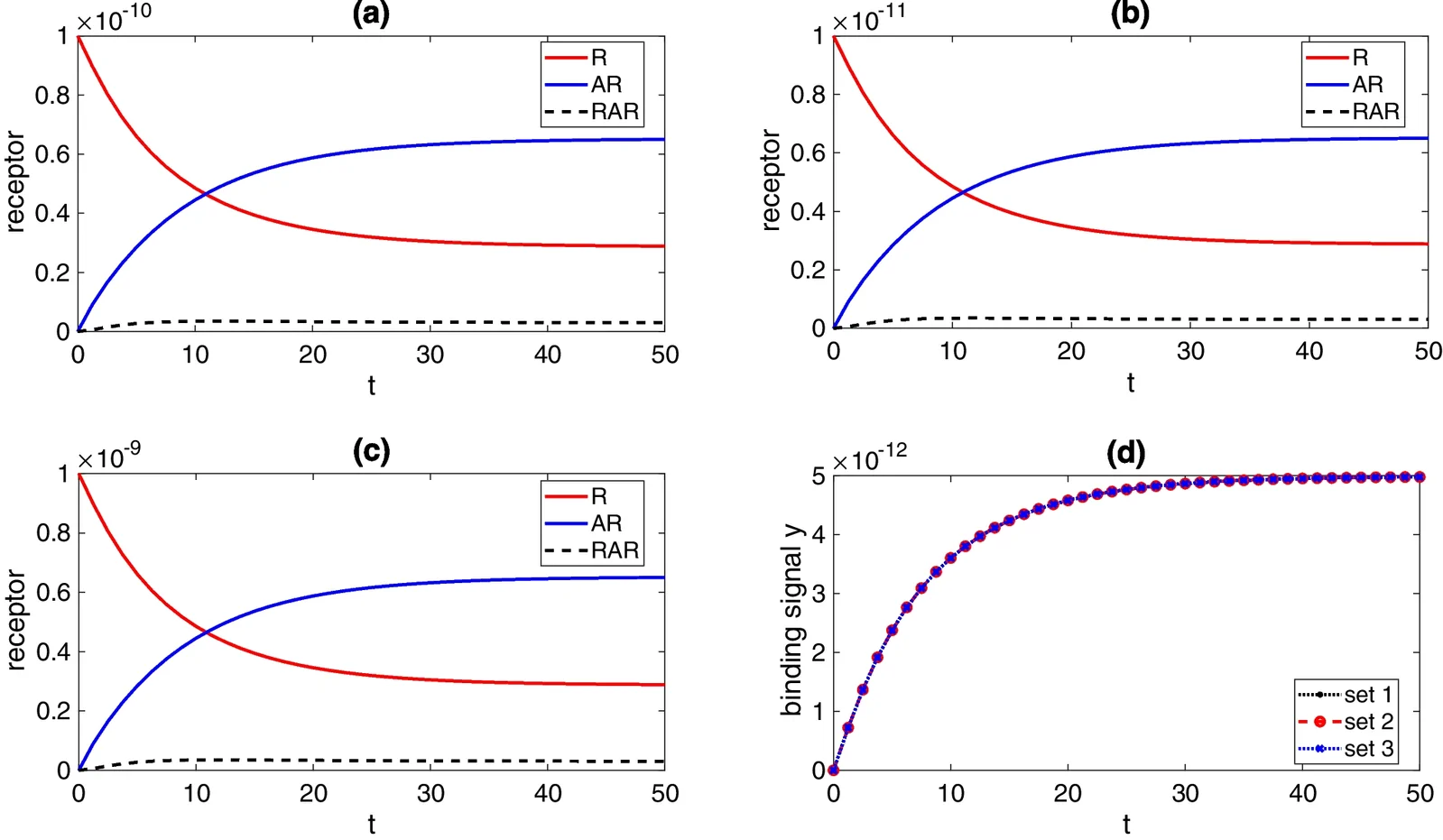
Non-identifiability for the ligand-induced dimerisation model demonstrated by showing identical signals for three different parameter sets, values for which are listed in Table 1. (a) Time courses for [*A*], [*AR*] and [*RAR*] for parameter set 1. (b) Time courses for [*A*], [*AR*] and [*RAR*] for parameter set 2. (c) Time courses for [*A*], [*AR*] and [*RAR*] for parameter set 3. (d) Time courses for measured signal *S = a([AR]+2[RAR])* for all three parameter sets. All three parameter sets give the same observed signal

The non-identifiability of parameters *a*, $R_{tot}$ and $\psi _{+}$ is illustrated in Fig. 3, where we show timecourses for the receptor species concentrations underlying the observed signal, together with the observed signal, for three different parameter sets (values for which are listed in Table 1). The underlying concentrations are different for each parameter set, but the signal is identical for all three sets.

**Table 1:** The parameter values for three different parameter sets are used to plot Fig. 3. The parameter combinations $aR_{tot}$ and are equal $\psi _+/a$ in each case

	Set 1	Set 2	Set 3
*a*	0.07	0.7	0.007
$k_{+}$	$8.4\times 10^6$	$8.4\times 10^6$	$8.4\times 10^6$
$k_{-}$	0.037	0.037	0.037
$\psi _+$	187	1870	18.7
$\psi _-$	26	26	26
$$R_{tot}$$	$$10^{-10} $$	$$10^{-11} $$	$$10^{-9} $$
$a R_{tot}$	$7\times 10^{-12}$	$7\times 10^{-12}$	$7\times 10^{-12}$
$\psi _+/a$	$2.67\times 10^{3}$	$2.67\times 10^{3}$	$2.67\times 10^{3}$

### Global structural identifiability when *a* is known

Although we found in Section“SIA for LID model - similarity transformation” that the system is not fully identifiable, we note that this is due only to the output being scaled, i.e., the signal *y* contains the factor *a*. Once the value of the experimental parameter *a* is known, the system becomes fully identifiable. Remarkably, this would then correspond to global identifiability for a single timecourse. This stands in contrast with the single-timecourse result for pre-dimerised GPCRs, for which no parameters are identifiable [43].

### Mitigating non-identifiability issues

We found in Section“SIA for LID model - similarity transformation” that, from a single time course, the parameters

5.48 $$\begin{aligned} k_{+},\quad k_{-},\quad \psi _-, \end{aligned}$$

in the LID model are identifiable. Furthermore, we find the identifiable parameter combination vector

5.49 $$\begin{aligned} \boldsymbol{\zeta }({\textbf {p}})=\begin{bmatrix} aR_{tot}\\ \psi _+R_{tot}\end{bmatrix}. \end{aligned}$$

In [44], the LID model is fitted to experimental data to estimate the model parameters. Therein, there are three sets of experiments, each with a different VEGF isoform. Within each of these sets, the experiments are repeated with five concentrations of VEGF. The quantity $R_{tot}$ and scaling factor *a* are the same in all experiment sets (as the cell membranes and measuring techniques are the same for each), while all other parameters may vary across experiments. In [43], it was shown that for pre-dimerised GPCR models, multiple timecourses corresponding to different ligand concentrations [*A*] could be used to achieve global identifiability, since identifiable parameter combinations depended on [*A*]. If we consider $\boldsymbol{\zeta }({\textbf {p}})$ as above, we note that *[A]* does not appear in any of the identifiable combinations, hence, using multiple concentrations of drug for the same experiment does not make any further parameters identifiable.

We note again, however, that the model will become fully identifiable from a single timecourse if the experimental apparatus-related factor *a* has been determined by other means.

## Discussion

Partially observed ODE systems (where not all of the state variables are observed experimentally) arise frequently in modelling of biological systems, and in mathematical pharmacology in particular. Quantitative pharmacology relies on the use of mathematical models in tandem with laboratory data and optimisation routines to estimate key parameters of pharmacological importance. Advances in the field of structural identifiability analysis (SIA) methodology have accompanied a rise in awareness of the issue of non-identifiability, but SIA considerations are largely absent from receptor theory studies. Here, building on the tutorial and new results in [45] for classical SIA methods applied to linear ligand-receptor binding models, we have presented a new analysis of nonlinear binding models of importance. The “classical” SIA methods of Taylor Series and Similarity Transformation extend naturally from linear models to nonlinear models, and have been applied here since they represent potentially practical approaches for low-dimensional nonlinear systems [46]. A priori SIA should become standard practice in ODE biomodelling studies where possible [10], and the current work meets the needs for SIA to be brought to a wide bio-audience [3] and for receptor theory to keep apace with SIA integration in systems biology.

Ligand excess, whereby ligand concentration is much greater than receptor concentration, is a common assumption in many pharmacological modelling studies, but consideration of ligand depletion is important in certain scenarios [12, 40]. Here we have considered extension of the linear ligand excess models for monomeric receptors and homodimerics receptor from [45] to the corresponding nonlinear models incorporating ligand depletion. The linear models are not globally structurally identifiable for a single timecourse (given by a single ligand concentration), and mitigating this issue involves extra experiments [45]. Remarkably, we have shown here that the corresponding nonlinear models are both globally structurally identifiable from a single timecourse. These new results may be useful in their own right when considering ligand depletion data [12, 40]. Furthermore, there is a potential practical application of these results in using parameter estimates obtained using depletion models as initial guesses for non-depletion model fitting. We propose a numerical study centred on this practical idea as future work.

Our new results for structural identifiability properties of ligand-induced dimerisation (LID) are of interest when considering the recent LID model analysis in [44]. Our SIA computations here are performed using the experimental aparatus-specific parameter *a* as an unknown. While the model is not globally structurally identifiable for any number of ligand concentrations under this assumption, three kinetic quantities of primary interest ($k_{+}$, $k_{-}$, $\psi _{-}$) are identifiable. Furthermore, if *a* is known then the model becomes fully identifiable from a single timecourse. This observation aligns with the guideline that scaling and offset parameters for experimental conditions need to be estimated in tandem with the ligand-receptor parameters [46]. Structural identifiability of the LID model is crucial towards quantifying and classifying the effects of cross-dimer cooperativity [44].

In deriving new SIA results, we have considered Taylor Series and Similarity Transformation approaches. The classical transfer function approach is not applicable to nonlinear systems. Similarly to the tractability results noted in [45], it is clear that, while theoretically intuitive, the Taylor Series approach becomes intractable for even for the low-dimensional nonlinear systems here. Of the possible nonlinear counterparts to the classical approaches considered in [45], it appears that only the nonlinear similarity transformation approach is analytically tractable for models of this complexity. Furthermore, the heavy, ad-hoc calculations in Sections“Ligand binding pre-dimerised receptor with ligand depletion” and“Ligand-induced dimerisation (LID)” suggest that this analytical approach would not be tractable for higher dimensional models in receptor theory or quantitative systems pharmacology (QSP).

In continuing to bring SIA to receptor theory, it is important to present the analytical approaches here, both as a natural extension of the existing linear analysis, and to demonstrate the borderline intractable nature of the method, where the systematic workflow is often spoiled by the heavy, symbolic, ad-hoc calculations required. This study has laid the groundwork for comparative computational implementations of SIA. Future work will now focus on computational (package) approaches to SIA for receptor theory models. The twofold purpose for this will be to (i) compare the computational speed and user-friendliness of new computational packages for the low-dimensional models already considered, and (ii) work towards a recommended computational template for SIA in higher dimensional ligand-receptor-signal transduction and systems biology models. Models of interest will range from kinetic operational models [13] to GPCR activation models [47]. Computational methods to be assessed include exact arithmetic rank [14] and singular value decomposition [32]. Of existing packages, Strike-GOLDD [7] and StrucID [31] are among the most promising [10, 46], and will form the basis for the study.

Our analysis has been limited to SIA, a theoretical property of a model and observed output. The SIA theory is now well established [10], and our analysis contributes important results, but further identifiability analysis challenges need to be addressed throughout mathematical pharmacology and QSP. Structural non-identifiability is often unavoidable for complex models, and there is debate over whether non-identifiable models should be avoided [28] due to the limitations this imposes on model predictions. Alternatively, consideration of both structural and practical identifiability issues and phenotype-guided filtering shows some potential towards the management of non-identifiability in the context of virtual populations for QSP models [48].

Beyond SIA, approaches to practical identifiability analysis (PIA) are important to understand, develop and implement, and have not been studied in the same level of detail [10, 46]. PIA considers not only the model itself, but the available data, and concerns the possibility of obtaining reliable parameter estimates in the presence of real-world data imperfections [27, 28, 48]. Wide application and development of PIA tools will be crucial in maximising the potential of state-of-the-art experimental techniques and mathematical modelling in receptor theory. Existing approaches include profile likelihood methods [20, 26, 27, 48]. Consideration of both SIA and PIA across QSP and mathematical pharmacology requires nontrivial effort but adds important results to new modelling studies [15]. A simple numerical parameter estimation experiment for the structurally identifiable monomeric receptor binding model with ligand depletion suggests that the degree of ligand depletion may affect practical identifiability properties of the system and hence the success of parameter estimation routines (see Appendix F). Therefore, our current work should be extended to include PIA as future work. Furthermore, within receptor theory, parameterisation of the Motulsky-Mahan model for two-ligand competition [23] is an example where PIA issues also need to be elucidated [8].

## Figures and Tables

**Fig. 4:**
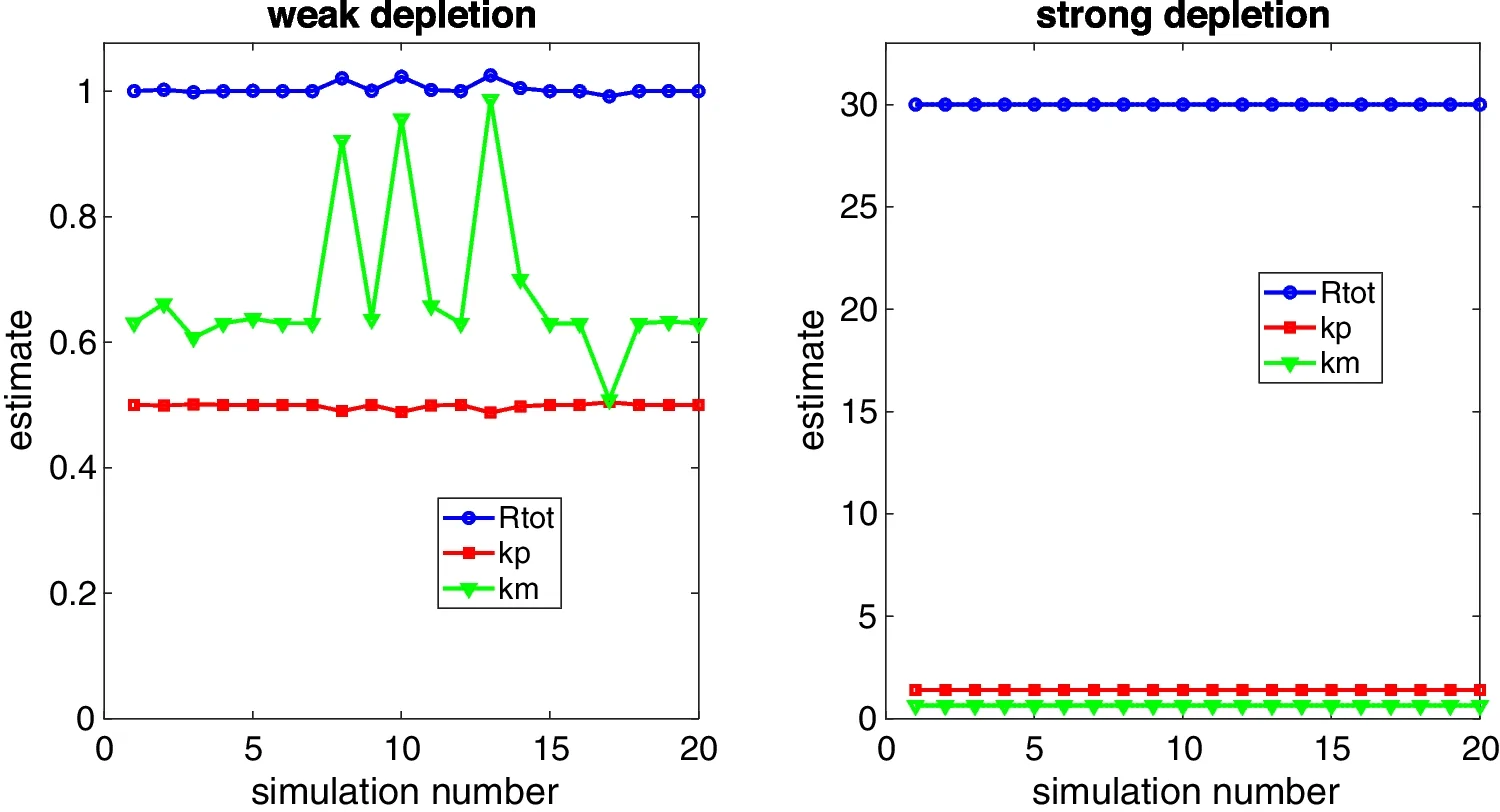
Parameter estimation results for monomeric receptor binding model Eq. 3.1 with ligand depletion. Left-hand plot is for weak depletion, with parameter values $$A_{tot}=30$$, $$R_{tot}=1$$, $$k_{+}=0.5$$, $$k_{-}=0.63$$. Right-hand plot is for strong depletion, with parameter values $$A_{tot}=1$$, $$R_{tot}=30$$, $$k_{+}=1.4$$, $$k_{-}=0.63$$. Each “simulation” involves the generation of pseudo-data using the exact solution Eq. 3.4 using 21 timepoints up to a maximum time of 0.4 for weak depletion or 0.3 for strong depletion, random noise at a relative level of $$10^{-6} \%$$ is added to the solution. Parameter estimates for $$R_{tot}$$, $$k_{+}$$ and $$k_{-} $$ are obtained for each simulation using randomised initial guesses and MATLAB’s fminsearch optimisation routine

## Data Availability

No datasets were generated or analysed during the current study.

## Appendix A The Lie bracket

The controllability matrix, as discussed in Section“Controllability and observability”, is constructed by use of the Lie bracket. We define this, as in [41]

### Definition A.1

The **Lie bracket** is an algebraic operation on two vector fields ${\textbf {f}}({\textbf {x}}), {\textbf {g}}({\textbf {x}}) \in \mathbb {R}^{n_x}$ that creates a third vector field $\mathcal {F}({\textbf {x}})$, which when taken with ${\textbf {g}}$ as the input control vector together with ${\textbf {u}}\in \mathbb {R}^{n_u}$ defines an embedding in $\mathbb {R}^{n_x}$ that maps the input to states. It is given as

A.1 $$\begin{aligned} (ad^1_{\textbf {f}},{\textbf {g}})=[{\textbf {f}},{\textbf {g}}]=\frac{\partial {\textbf {g}}({\textbf {x}})}{\partial {\textbf {x}}} {\textbf {f}}({\textbf {x}})-\frac{\partial {\textbf {f}}({\textbf {x}})}{\partial {\textbf {x}}} {\textbf {g}}({\textbf {x}}), \end{aligned}$$

where $(ad^1_{\textbf {f}},{\textbf {g}})$ is the adjoint operator, with the superscript 1 denoting the order of the Lie bracket [41]. Higher order brackets are calculated recursively as

A.2 $$\begin{aligned} (ad^2_{\textbf {f}},{\textbf {g}})&=[{\textbf {f}},[{\textbf {f}},{\textbf {g}}]]=\frac{\partial (ad^1_{\textbf {f}},{\textbf {g}})}{\partial {\textbf {x}}} {\textbf {f}}({\textbf {x}})-\frac{\partial {\textbf {f}}({\textbf {x}})}{\partial x}(ad^1_{\textbf {f}},{\textbf {g}}), \nonumber \\&\hspace{1cm}\vdots \\ (ad^k_{\textbf {f}},{\textbf {g}})&=[{\textbf {f}},(ad^{k-1}_{\textbf {f}},{\textbf {g}})]. \nonumber \end{aligned}$$

## Appendix B The Lie derivative

To construct the observability matrix, as discussed in Section“Controllability and observability”, we use the Lie bracket, which we define as in [38]:

### Definition B.1

The **Lie derivative** of ${\textbf {f}}({\textbf {x}})$ with respect to ${\textbf {y}}({\textbf {x}})$ is given as

B.1 $$\begin{aligned} L^1_{\textbf {f}} {\textbf {y}}({\textbf {x}})=\frac{\partial {\textbf {y}}({\textbf {x}})}{\partial {\textbf {x}}} {\textbf {f}}({\textbf {x}}), \end{aligned}$$

with higher order derivatives being calculated recursively as

B.2 $$\begin{aligned} L^2_{\textbf {f}} {\textbf {y}}({\textbf {x}})&=\dfrac{\partial L^1_{\textbf {f}} {\textbf {y}}({\textbf {x}})}{\partial {\textbf {x}}} {\textbf {f}}({\textbf {x}}), \nonumber \\&\vdots \\ L^i_{\textbf {f}} {\textbf {y}}({\textbf {x}})&=\dfrac{\partial L^{i-1}_{\textbf {f}} {\textbf {y}}({\textbf {x}})}{\partial {\textbf {x}}} {\textbf {f}}({\textbf {x}}), \nonumber \end{aligned}$$

## Appendix C Linear equivalence

To determine identifiability of a linear ODE system, we use the algebraic equivalence theorem [9, 29], which states that

### Theorem C.1

(*F*, *G*, *H*) is algebraically equivalent to $(\widetilde{F},\widetilde{G},\widetilde{H})$ if and only if there exists a continuously differentiable matrix $T:\mathbb {R}^{n_x}\rightarrow \mathbb {R}^{n_x}$, such that

C.1a $$\begin{aligned}& \qquad \qquad \qquad \qquad \det T \ne 0, \qquad \qquad \qquad \qquad \end{aligned}$$

C.1b $$\begin{aligned}&\qquad \qquad \qquad \qquad T\widetilde{{\textbf {x}}}_0 \qquad = {\textbf {x}}_0, \qquad \qquad \qquad \qquad \end{aligned}$$

C.1c $$\begin{aligned}& \qquad \qquad \qquad \qquad T\widetilde{F}\qquad =FT, \end{aligned}$$

C.1d $$\begin{aligned}&\qquad \qquad \qquad \qquad \qquad \,\, T\widetilde{G}\qquad =G, \end{aligned}$$

C.1e $$\begin{aligned}&\qquad \qquad \qquad \qquad \qquad \quad \widetilde{H}\qquad =HT, \end{aligned}$$

where $$\widetilde{F}=F(\widetilde{{\textbf {p}}})$$, $$\widetilde{G}=G(\widetilde{{\textbf {p}}})$$ and $$\widetilde{H}=H(\widetilde{{\textbf {p}}})$$.

## Appendix D Nonlinear equivalence

Identifiability of a nonlinear ODE system is determined via the local state isomorphism theorem, given as (as in [4, 5, 35]):

### Theorem D.1

Assume that the model of Eq. 2.1 is locally reduced at ${\textbf {x}}({\textbf {p}})$ for all ${\textbf {p}}\in \Omega$. Consider the parameter values ${\textbf {p}}, \widetilde{{\textbf {p}}}\in \Omega$, an open neighbourhood *V* of ${\textbf {x}}_0(\widetilde{{\textbf {p}}})$ in *M* (a subset of $\mathbb {R}^{n_x}$ containing the initial state), and any analytical mapping $\lambda :V\rightarrow \mathbb {R}^{n_x}, V\subset \mathbb {R}^{n_x}$ such that

D.1a $$\begin{aligned}&(i) \qquad \qquad \qquad \text {rank}\left( \dfrac{\partial \lambda (\widetilde{{\textbf {x}}})}{\partial \widetilde{{\textbf {x}}}}\right) \qquad = n_x,\quad \forall \widetilde{{\textbf {x}}}\in V, \end{aligned}$$

D.1b $$\begin{aligned}&(ii)\qquad \qquad \qquad \qquad \lambda (\widetilde{{\textbf {x}}}_0({\textbf {p}})) \qquad = {\textbf {x}}_0(\widetilde{{\textbf {p}}}), \end{aligned}$$

D.1c $$\begin{aligned}&(iii) \qquad \qquad \qquad \,\, f(\lambda (\widetilde{{\textbf {x}}}),{\textbf {p}}) \qquad =\dfrac{\partial \lambda (\widetilde{{\textbf {x}}})}{\partial \widetilde{{\textbf {x}}}}f(\widetilde{{\textbf {x}}},\widetilde{{\textbf {p}}}), \end{aligned}$$

D.1d $$\begin{aligned}&\qquad \qquad \qquad \qquad \,\,\, g(\lambda (\widetilde{{\textbf {x}}}),{\textbf {p}})\qquad =\dfrac{\partial \lambda (\widetilde{{\textbf {x}}})}{\partial \widetilde{{\textbf {x}}}}g(\widetilde{{\textbf {x}}},\widetilde{{\textbf {p}}}), \end{aligned}$$

D.1e $$\begin{aligned}&\qquad \qquad \qquad \qquad \,\,\, h(\lambda (\widetilde{{\textbf {x}}}),{\textbf {p}})\qquad =h(\widetilde{{\textbf {x}}},\widetilde{{\textbf {p}}}), \end{aligned}$$

for all $\widetilde{{\textbf {x}}}\in V$. Then the system in Eq. 2.1 is globally identifiable at ${\textbf {p}}$ if and only if Eqs. 2.16a-eq:siaspsnonlinspscond5 implies $\widetilde{{\textbf {p}}}={\textbf {p}}$.

## Appendix E SIA for LID model - Taylor series

We first apply the Taylor series method that we introduced in Section“Taylor series method” to the LID model Eq. 5.1. Clearly, $n_{x}=3$, but the system is nonlinear and so it is unknown how many coefficients need to be determined for identifiability properties to be established.

The Taylor series for *y* about *t=0* is

E.1 $$\begin{aligned} y(t)=y(0)+ty^{(1)}(0)+\frac{t^2}{2!}y^{(2)}(0)+... \; , \end{aligned}$$

where bracketed superscript is used to indicate the order of the derivative with respect to *t*. The first coefficient is simply

E.2 $$\begin{aligned} y(0)=0. \end{aligned}$$

The first derivative of the output function is found (using Eq. 5.1) to be

E.3 $$\begin{aligned} y^{(1)}&=a\left\{\begin{array}{c} \Big (k_{+}[A][R]- k_{-}[AR]- \psi _+k_{+}[R][AR]+ \psi _-k_{-}[RAR]\Big )\\+2\Big (\psi _+k_{+}[R][AR]- \psi _-k_{-}[RAR]\Big )\end{array} \right\} \nonumber \\&=a\Big (k_{+}[A][R]- k_{-}[AR]+ \psi _+k_{+}[R][AR]- \psi _-k_{-}[RAR]\Big ). \end{aligned}$$

After substituting *t=0* and using the initial conditions, we obtain the unique coefficient corresponding to the first derivative as

E.4 $$\begin{aligned} y^{(1)}(0) = ak_{+}[A]R_{tot}. \end{aligned}$$

Clearly, the quantity $ak_{+}R_{tot}$ is an identifiable parameter combination.

The second coefficient is found by differentiating Eq. E.3 and again substituting *t=0*. We find that

E.5 $$\begin{aligned} y^{(2)}(0) = -ak_{+}[A]R_{tot}(k_{+}[A]+k_{-}-\psi _+k_{+}R_{tot}). \end{aligned}$$

In view of Eq. E.4, we find that the second identifiable parameter combination is

$$\begin{aligned} k_{+}[A]+k_{-}-\psi _+k_{+}R_{tot}. \end{aligned}$$

We continue to calculate coefficients using repeated differentiation and substitution of *t=0*. This yields

E.6 $$\begin{aligned} y^{(3)}(0)&=ak_{+}[A]R_{tot}(k_{+}^2[A]^2+2k_{+}k_{-}[A]+k_{-}^2-4\psi _+k_{+}^2[A]R_{tot}\nonumber \\&\quad -\psi _+^2k_{+}^2R_{tot}^2-\psi _+\psi _-k_{+}k_{-}R_{tot}), \end{aligned}$$

E.7 $$\begin{aligned} y^{(4)}(0)&=a k_{+}[A]R_{tot}(\psi _+^3 k_{+}^3 R_{tot}^3 + 2 \psi _+^2 \psi _-k_{+}^2 k_{-}R_{tot}^2 \nonumber \\&\quad+ 3 \psi _+^2 k_{+}^3 [A]R_{tot}^2 + \psi _+^2 k_{+}^2 k_{-}R_{tot}^2 + \psi _+\psi _-^2 k_{+}k_{-}^2 R_{tot}\nonumber \\&\quad+ 4 \psi _+\psi _-k_{+}^2 k_{-}[A]R_{tot}+ 11 \psi _+k_{+}^3 [A]^2 R_{tot}\nonumber \\&\quad+ 6 \psi _+k_{+}^2 k_{-}[A]R_{tot}- \psi _+k_{+}k_{-}^2 R_{tot}- k_{+}^3 [A]^3 \nonumber \\&\quad- 3 k_{+}^2 k_{-}[A]^2 - 3 k_{+}k_{-}^2 [A]- k_{-}^3), \end{aligned}$$

E.8 $$\begin{aligned} y^{(5)}(0)&=-a k_{+}[A]R_{tot}(\psi _+^4 k_{+}^4 R_{tot}^4 + 3 \psi _+^3 \psi _-k_{+}^3 k_{-}R_{tot}^3\nonumber \\&\quad - 4 \psi _+^3 k_{+}^4 [A]R_{tot}^3 + 2 \psi _+^3 k_{+}^3 k_{-}R_{tot}^3 + 3 \psi _+^2 \psi _-^2 k_{+}^2 k_{-}^2 R_{tot}^2\nonumber \\&\quad + 6 \psi _+^2 \psi _-k_{+}^3 k_{-}[A]R_{tot}^2 + 2 \psi _+^2 \psi _-k_{+}^2 k_{-}^2 R_{tot}^2\nonumber \\&\quad - 2 \psi _+^2 k_{+}^4 [A]^2 R_{tot}^2 + 18 \psi _+^2 k_{+}^3 k_{-}[A]R_{tot}^2 \nonumber \\&\quad+ \psi _+\psi _-^3 k_{+}k_{-}^3 R_{tot}+ 4 \psi _+\psi _-^2 k_{+}^2 k_{-}^2 [A]R_{tot}[A]^2 R_{tot} \nonumber \\&\quad+ 11 \psi _+\psi _-k_{+}^3 k_{-}+ 6 \psi _+\psi _-k_{+}^2 k_{-}^2 [A]R_{tot}- \psi _+\psi _-k_{+}k_{-}^3 R_{tot}\nonumber \\&\quad+ 26 \psi _+k_{+}^4 [A]^3 R_{tot} + 34 \psi _+k_{+}^3 k_{-}[A]^2 R_{tot}\nonumber \\&\quad+ 6 \psi _+k_{+}^2 k_{-}^2 [A]R_{tot}- 2 \psi _+k_{+}k_{-}^3 R_{tot}- k_{+}^4 [A]^4 - 4 k_{+}^3 k_{-}[A]^3 \nonumber \\&\quad - 6 k_{+}^2 k_{-}^2 [A]^2 - 4 k_{+}k_{-}^3 [A]- k_{-}^4). \end{aligned}$$

There is not a clear systematic approach for using the expressions above to determine identifiability. For higher order derivatives in the Taylor expansion, we expect the expressions to be even more unwieldy, and any progress would rely on an ad hoc approach and lengthy symbolic computation. These issues were also noted in [4]. At this point, we choose not to pursue the Taylor series method for the LID model.

## Appendix F Weak and strong ligand depletion simulations

In Fig. 4, we show the results of 20 runs of a parameter estimation code applied to the monomeric receptor model Eq. 3.1 for cases of weak and strong ligand depletion. The code fits the model to pseudo-data (a simulation result plus random noise) for a single timecourse. In the case of strong depletion, where $\frac{A_{tot}}{R_{tot}}\ll 1$, we note that the resulting estimates for the parameters $k_{+}$, $k_{-}$ and $R_{tot}$ show relatively little variation from one optimisation run to the next (see Fig. 4 right-hand plot). For the case of weak depletion, where $\frac{A_{tot}}{R_{tot}}\gg 1$, there is relatively more variation in the individual parameters (see Fig. 4 left-hand plot). This simple numerical experiment hints at issues of practical non-identifiability which need to be explored in future work.
